# Site-specific immobilization of the endosialidase reveals QSOX2 is a novel polysialylated protein

**DOI:** 10.1093/glycob/cwae026

**Published:** 2024-03-15

**Authors:** Carmanah Hunter, Tahlia Derksen, Sogand Makhsous, Matt Doll, Samantha Rodriguez Perez, Nichollas E Scott, Lisa M Willis

**Affiliations:** Department of Biological Sciences, University of Alberta, 116 St & 85 Ave, Edmonton, AB, T6G 2R3, Canada; Department of Biological Sciences, University of Alberta, 116 St & 85 Ave, Edmonton, AB, T6G 2R3, Canada; Department of Biological Sciences, University of Alberta, 116 St & 85 Ave, Edmonton, AB, T6G 2R3, Canada; Department of Biological Sciences, University of Alberta, 116 St & 85 Ave, Edmonton, AB, T6G 2R3, Canada; Department of Biological Sciences, University of Alberta, 116 St & 85 Ave, Edmonton, AB, T6G 2R3, Canada; Department of Microbiology and Immunology, University of Melbourne, Melbourne, VIC 3000, Australia; Department of Biological Sciences, University of Alberta, 116 St & 85 Ave, Edmonton, AB, T6G 2R3, Canada; Department of Medical Microbiology and Immunology, University of Alberta, 116 St & 85 Ave, Edmonton, AB, T6G 2R3, Canada

**Keywords:** biotinylation, EndoN, protein immobilization, proteomics, sialic acid

## Abstract

Polysialic acid (polySia) is a linear polymer of α2,8-linked sialic acid residues that is of fundamental biological interest due to its pivotal roles in the regulation of the nervous, immune, and reproductive systems in healthy human adults. PolySia is also dysregulated in several chronic diseases, including cancers and mental health disorders. However, the mechanisms underpinning polySia biology in health and disease remain largely unknown. The polySia-specific hydrolase, endoneuraminidase NF (EndoN), and the catalytically inactive polySia lectin EndoN_DM_, have been extensively used for studying polySia. However, EndoN is heat stable and remains associated with cells after washing. When studying polySia in systems with multiple polysialylated species, the residual EndoN that cannot be removed confounds data interpretation. We developed a strategy for site-specific immobilization of EndoN on streptavidin-coated magnetic beads. We showed that immobilizing EndoN allows for effective removal of the enzyme from samples, while retaining hydrolase activity. We used the same strategy to immobilize the polySia lectin EndoN_DM_, which enabled the enrichment of polysialylated proteins from complex mixtures such as serum for their identification via mass spectrometry. We used this methodology to identify a novel polysialylated protein, QSOX2, which is secreted from the breast cancer cell line MCF-7. This method of site-specific immobilization can be utilized for other enzymes and lectins to yield insight into glycobiology.

## Introduction

Polysialic acid (polySia) is a linear polymer of α2,8-linked sialic acid residues that is of fundamental biological interest due to its pivotal roles in the regulation of the nervous, immune, and reproductive systems in healthy human adults. PolySia extends both *N*- and *O*-linked glycans on a small number of proteins and imparts profound consequences on the proteins and cells to which it is attached (Nakata and Troy 2nd 2005; Colley et al. 2014; Schnaar et al. 2014). Cells containing polysialylated proteins are typically more migratory, and polySia is required for axonal migration (El Maarouf and Rutishauser 2003), neurite outgrowth (Franceschini et al. 2001), and dendritic cell migration (Rey-Gallardo et al. 2010). PolySia also acts as an immune checkpoint by attenuating the immune response (Curreli et al. 2007; Stamatos et al. 2014; Karlstetter et al. 2017), similar to what has been observed for other sialosides (Adams et al. 2018; Barenwaldt and Laubli 2019). In addition to its critical roles in healthy humans, dysregulation of polySia contributes to the pathology of several chronic diseases, including mental health disorders, neurodegenerative and autoimmune diseases, and cancer (Mikkonen et al. 1999; Tanaka et al. 2000; Daniel et al. 2001; Suzuki et al. 2005; Yoshimi et al. 2005; Strekalova et al. 2006; McAuley et al. 2009; Anney et al. 2010; Heimburg-Molinaro et al. 2011; Falconer et al. 2012; Varea et al. 2012; Maheu et al. 2013; Piras et al. 2015; Yang et al. 2015; Gong et al. 2017; Fullerton et al. 2018; Khan et al. 2023). However, while some progress has been made into the mechanistic details supporting polySia biology, many have yet to be elucidated.

While there are several tools with which to detect and manipulate polySia, investigating its biology remains a challenge. The α-polySia antibody mAb735 is both sensitive and specific for polySia (Häyrinen et al. 2002). However, the large, anionic nature of the polymer makes identification of polysialylated proteins via pull down approaches challenging (Werneburg et al. 2016). Many proteins, particularly basic proteins, tend to interact non-specifically with polySia and cannot be removed under the mild washing conditions of immunoprecipitation. The resulting proteomics experiments often contain hundreds of hits which require either a substantial number of antibodies for validation or a biased selection of hits to validate based on known function and subcellular localization (Curreli et al. 2007; Simon et al. 2013; Kiermaier et al. 2016; Werneburg et al. 2016).

Another tool that has been used extensively to study polySia is the endoneuraminidase NF (EndoN). EndoN is a tailspike endohydrolase protein from a bacteriophage that infects the polySia capsule-containing *Escherichia coli* K1 (Pelkonen et al. 1992). EndoN is a trimer with both polySia-binding and polySia-hydrolysis functions, which normally allows the bacteriophage to penetrate the capsule layer to access the bacterial cell surface (Pelkonen et al. 1992). Heterologous expression of EndoN in *E. coli* yields a heat- and detergent-stable protein that can be used to specifically hydrolyze polySia (Jokilammi et al. 2004). EndoN has been used to demonstrate the role of polySia in phagocytosis (Stamatos et al. 2014) and activation of T cells (Curreli et al. 2007), among many others. Furthermore, endosialidases were some of the original hydrolases converted to lectins through mutation of key active site residues (Pelkonen et al. 1992). The EndoN double mutant (EndoN_DM_) is a catalytically inactive version of EndoN that binds polySia with a K_D_ of ~10^−8^ M (Jokilammi et al. 2004). Fusion of the EndoN_DM_ lectin with green fluorescent protein yielded a robust new tool complementary to mAb735 which could be used to visualize polySia by microscopy and flow cytometry (Jokilammi et al. 2004).

We hypothesized that site-specific immobilization of EndoN and EndoN_DM_ could improve their usefulness in studying polySia biology. Here, we report engineering EndoN and EndoN_DM_ to contain a 15-amino acid Avi tag, which can be efficiently biotinylated and then immobilized on streptavidin-coated agarose or magnetic beads. Efficient removal of EndoN post-hydrolysis has long been an issue in the field and we show that immobilization of EndoN on magnetic beads significantly improves the efficiency with which the enzyme is removed from samples, while retaining its ability to hydrolyze polySia. Efficient removal of enzyme post-hydrolysis is necessary to properly investigate interactions between multiple polysialylated species, as in the case of dendritic cell – T cell interactions. We also show that immobilized EndoN_DM_ can be used to enrich polysialylated proteins from complex mixtures, even in the presence of detergent. The enriched mixture can then be used to identify polysialylated proteins by mass spectrometry. We demonstrate that the NK-92 cell line and human serum both contain polysialylated NCAM and ST8Sia4. We then used the methodology to discover that QSOX2 is a novel polysialylated protein secreted from the breast cancer cell line MCF-7.

## Results

### EndoN is heat stable and remains associated with cells after washing

**Fig. 1:**
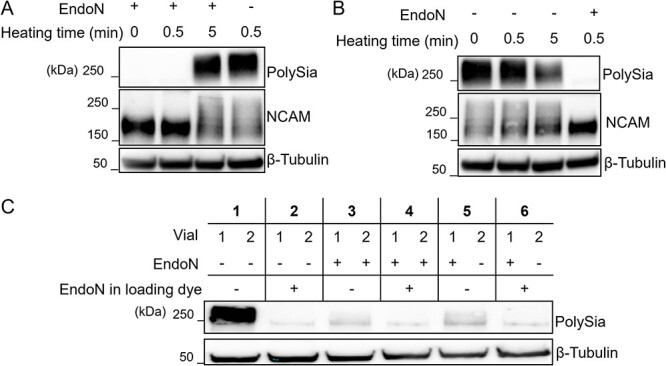
EndoN is heat stable and remains associated with the NK-92 cells after washing. A) Immunoblot of NK-92 cell lysate treated with soluble EndoN that had been pre-heated at 95 °C for 0.5 or 5 min. After mixing the lysate with either control or heat-treated EndoN in SDS-PAGE sample buffer, all samples were heated at 95 °C for 30 sec to denature proteins. B) Immunoblot of NK-92 cell lysate heated at 95 °C for 0.5 or 5 min in SDS-PAGE sample buffer. In the sample containing EndoN, soluble EndoN was added to the lysis buffer before heating. C) Samples containing 10^6^ NK-92 cells were incubated with either PBS or soluble EndoN for 30 min at 37 °C. After washing the cells 3 times with PBS, cells were mixed as indicated and then incubated at 37 °C for 1 h. After making lysates, equivalent amounts of protein was loaded in each well. As an additional control for polySia hydrolysis, a portion of each sample was treated with soluble EndoN in the loading dye. The experiments were performed in biological triplicate.

In experiments designed to investigate the role of polySia in cell-cell interactions, there are three possible sources of polySia: cell type 1, cell type 2, and media containing fetal bovine serum. We found that it was not possible to remove polySia from one of these components without compromising the rest of the experiment. For example, to remove polySia from media, we had proposed to treat media with EndoN and then inactivate the enzyme by heating. However, EndoN was sufficiently heat-stable that the length of time required to inactivate it also decreased the amount of polySia in the control (untreated) samples. As serum is challenging to blot due to the high concentration of albumin, we demonstrated this phenomenon in lysates from NK-92 cells (Fig. 1A and B). These cells represent a natural killer cell line derived from a 50 year old male with rapidly progressive non-Hodgkin's lymphoma and make polysialylated NCAM. Additionally, it was not possible to entirely remove EndoN from intact cells. NK-92 cells were treated with EndoN to remove cell surface polySia and then cells were washed three times in PBS, the standard method for washing cells. However, when these washed cells were mixed 1:1 with polysialylated (untreated) NK-92 cells, the majority of polySia in the mixture was hydrolyzed, indicating residual EndoN was not removed with washing (Fig. 1C).

### Immobilization of EndoN and EndoN_DM_ on streptavidin beads

**Fig. 2:**
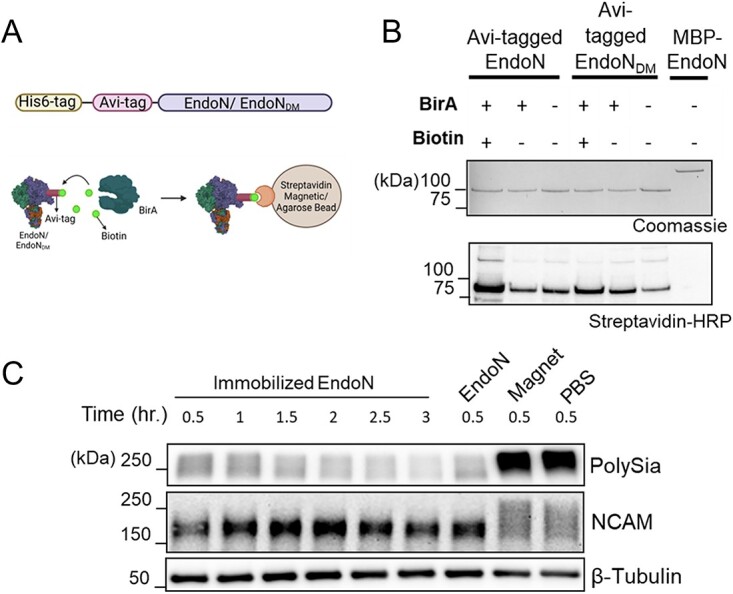
Immobilization of EndoN and EndoN_DM_. A) Schematic figure of Avi-tagged proteins and workflow for biotinylation and immobilization. B) Coomassie gel (top panel) and streptavidin-HRP blot (bottom panel) of the purified recombinant proteins after the biotinylation reaction. MBP-EndoN was used as non-Avi-tagged control. 0.25 μg protein from each reaction was loaded onto the gel. C) The activity of the immobilized enzyme was assessed by incubating intact NK-92 cells with magnet-conjugated EndoN at 37 °C. Cells were collected, washed, and lysed every 0.5 h for 3 h. As a control, intact NK-92 cells were incubated with soluble EndoN (EndoN), unconjugated magnetic beads (magnet) or PBS for 0.5 h. 7 μg protein of each sample was loaded on the gel. The experiments were performed in biological triplicate.

We hypothesized that immobilization of EndoN would overcome the challenge of completely removing the protein after hydrolysis of polySia in biological samples. To immobilized EndoN, we chose to use a genetically encoded Avi tag, which can be biotinylated using the BirA biotin ligase from *E. coli* (Fig. 2A) (Li and Sousa 2012). The near-covalent interaction between biotin and streptavidin would result in an immobilized protein that was stable, even under strong washing conditions. In parallel, we also prepared an immobilized version of the EndoN_DM_ protein for potential use in isolating polysialylated proteins.

His_6_-Avi-tagged proteins were expressed in *E. coli* and purified using immobilized metal affinity chromatography (Fig. S1), followed by biotinylation using recombinant BirA (Figs 2B and S2). Since BirA is an *E. coli* protein, we observed some endogenous biotinylation, which is absent in the non-Avi-tagged version of EndoN. However, in vitro addition of BirA substantially increased the amount of biotinylated EndoN/EndoN_DM_.

After biotinylation, EndoN and EndoN_DM_ were immobilized on streptavidin-coated magnetic or agarose beads respectively (Fig. S3). Immobilized EndoN retained its ability to hydrolyse polySia in NK-92 cell lysates (Fig. 2C). We reasoned that washing the beads with 1% SDS after immobilizing the proteins would remove all loosely associated material, thereby ensuring that EndoN would not wash off the beads in subsequent experiments. Washing the immobilized EndoN with SDS did not substantially decrease polySia hydrolase activity (Fig. S4). Additionally, we examined the stability of the immobilized EndoN and found that the beads could be reused on subsequent days without sacrificing activity (Fig. S5).

### Immobilized EndoN is effectively removed from cells

**Fig. 3:**
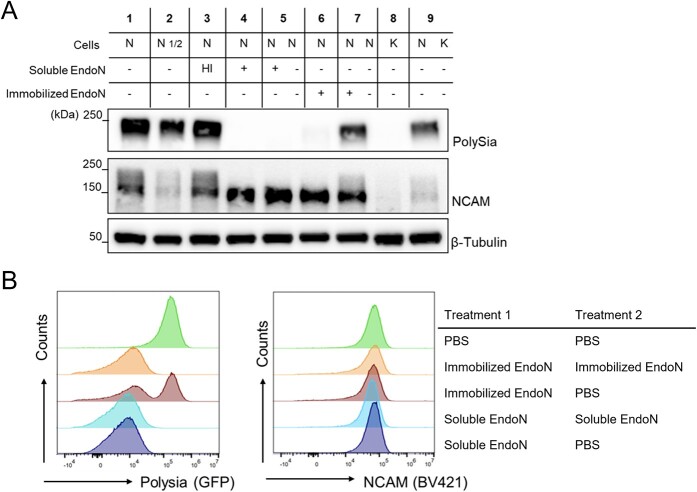
Magnet-conjugated EndoN can be efficiently removed from cells. Cells were treated with soluble EndoN, immobilized EndoN, or PBS. After washing, different permutations of cells were mixed in a 1:1 ratio, incubated at 37 °C for 1 h and then analyzed by immunoblotting (A) or flow cytometry (B). For immunoblotting, N and K correspond to NK-92 and K562 cells respectively. 10 μg lysate was loaded in all wells except N_1/2_, which contained 5 μg. In lanes with two letters in the cell row, cells were treated separately with the indicated conditions before mixing together. In lanes with one letter in the label, the cells that were mixed were from identical treatment conditions. HI corresponds to heat inactivated EndoN. All flow cytometry experiments were performed with NK-92 cells. All experiments were performed in at least biological triplicate.

To demonstrate that immobilized EndoN was effectively removed from cells, we performed mixing experiments where two vials of cells were treated separately with or without EndoN and then mixed together and analyzed by immunoblotting (Figs 3A and S6). To facilitate removal of immobilized EndoN, we immobilized the enzyme on magnetic beads, which could be removed using a standard magnet. If we effectively removed EndoN from cells after hydrolysis, any polySia added subsequently should remain intact. Similar to what we observed in Fig. 1, mixing NK-92 cells treated with soluble EndoN and NK-92 cells treated with PBS results in a complete loss of polySia due to residual EndoN sticking to cells (Fig. 3A, Lane 5). In contrast, we observed a roughly 50% decrease in polySia signal after mixing NK-92 cells treated with immobilized EndoN and NK-92 cells treated with PBS (Fig. 3A, Lane 7) compared to controls treated with PBS or heat-inactivated EndoN (Lanes 1 and 3 respectively). This ~50% reduction is comparable to the signal observed for half the amount of PBS-treated NK-92 lysate (Lane 2) as well as NK-92 cells mixed 1:1 with the non-polysialylated K562 cell line (Lane 9).

To corroborate our immunoblotting results, we analyzed the mixed cell populations by flow cytometry (Figs 3B and S7). Again, we observed a complete loss of polySia after mixing soluble EndoN-treated cells with PBS-treated cells, as indicated by the single polySia population, supporting the observation that EndoN is not removed by washing. However, mixing immobilized EndoN-treated cells with PBS-treated cells yielded both polySia^−^ and polySia^+^ populations, as we would expect if EndoN was properly removed. In all cases, NCAM signal was unaffected, suggesting that the changes in polySia are not due to changes in cell surface expression of NCAM.

### Immobilized EndoN_DM_ can be used to isolate polysialylated proteins for identification by proteomics

**Fig. 4:**
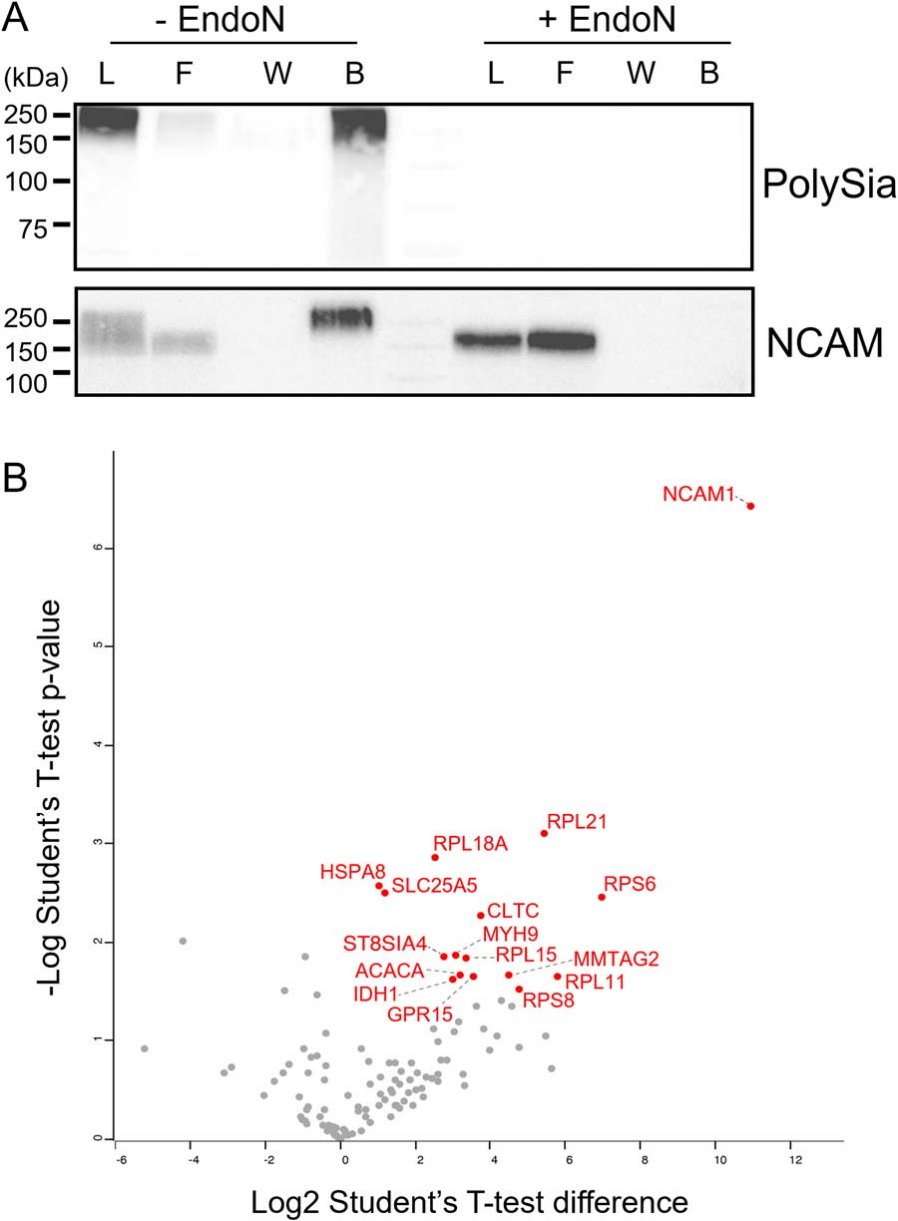
Proteomic analysis of polysialylated proteins from NK-92 cells. A) Immunoblot analysis of NK-92 cell lysate samples treated with PBS or with active EndoN before isolation on immobilized EndoN_DM_. α-polySia and α-NCAM blots show lysate (L), flowthrough (F), wash (W), and boiled beads (B). B) Volcano plot of proteins identified in samples relative to those pretreated with active EndoN. Proteins with a significant difference are labelled. The known polysialylated proteins NCAM1 and ST8Sia4 are highlighted (*n* = 4 per group, assessed with student’s T-test).

The current method for identifying novel polysialylated proteins is immunoprecipitation, which suffers from low sensitivity and specificity (Curreli et al. 2007; Simon et al. 2013; Kiermaier et al. 2016; Werneburg et al. 2016). We hypothesized that immobilized EndoN_DM_ could provide a better strategy as it binds polySia almost as well as the α-polySia mAb735 but is detergent resistant and may allow for washing with harsh conditions to remove non-specifically bound proteins. To validate that polySia binds to immobilized EndoN_DM_, we incubated beads with NK-92 cell lysates, washed them with 1% SDS, and then followed polySia and NCAM by immunoblotting (Fig. 4A). The majority of the polySia remained associated with the beads, with very little in the flow-through and none in the wash, indicating that the association between polySia and EndoN_DM_ is stable to harsh washing conditions. Similarly, only high molecular weight NCAM (i.e. polysialylated) remained associated with the beads, while the lower (non-polysialylated) band was lost in the flow-through, indicating that the association of protein with immobilized EndoN_DM_ is polySia-dependent. As a negative control, the NK-92 cell lysate was pretreated with EndoN before isolation. We observed no signal corresponding to NCAM in the EndoN-treated sample bound to the beads, confirming the specificity of the interaction.

To determine whether immobilized EndoN_DM_ could be used to isolate polysialylated proteins for identification by mass spectrometry, we incubated immobilized EndoN_DM_ with NK-92 lysates. For comparison, we also incubated immobilized EndoN_DM_ with NK-92 lysates pretreated with EndoN. Proteomic analysis of these samples yielded a 10-fold enrichment of NCAM, confirming our ability to enrich polysialylated proteins (Fig. 4B, Supplementary Table 1). Additionally, we also saw enrichment of the polysialyltransferase ST8Sia4, which is known to autopolysialylate, though it was only detected in three of the four replicates. The remaining few enriched proteins consisted mainly of ribosomal proteins, which frequently contaminate polySia isolations.

### Identification of polysialylated NCAM and ST8Sia4 in serum

**Fig. 5:**
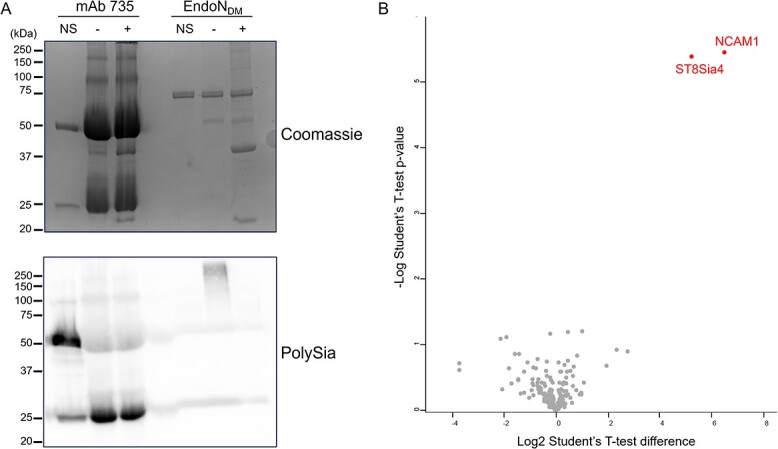
Analysis of polysialylated proteins from human serum. A) SDS-PAGE and α-polySia immunoblot of immunoprecipitation using mAb 735 and pull-down using immobilized EndoN_DM_. Negative controls include no added serum (NS) and pretreatment of the samples with EndoN (+). C) Volcano plot of proteins identified in samples relative to those pretreated with active EndoN (*n* = 4 per group, assessed with student’s T-test). Proteins with a significant difference are labelled. Samples were analyzed in biological quadruplicates.

To probe whether our methodology could be useful in a more complex sample, we investigated whether we could isolate polysialylated proteins from human serum. Serum contains very high concentrations of albumin (40 g/L) and antibodies (up to 20 g/L) (Jazayeri et al. 2013), which complicate immunoprecipitation and immunoblotting analysis. We compared the abilities of protein G-bound mAb735 and immobilized EndoN_DM_ to isolate polysialylated proteins from human serum. We observed a huge increase in total protein immunoprecipitated from serum using mAb735, as measured by SDS-PAGE (Fig. 5A). However, the same proteins were also present in samples pretreated with EndoN, suggesting that none is polysialylated. Additionally, polySia could not be detected in an α-polySia immunoblot (Fig. 5B). In comparison, we observed very few enriched proteins after incubating immobilized EndoN_DM_ with serum and were able to detect polySia in an immunoblot (Fig. 5A and B). These results encouraged us to use the immobilized EndoN_DM_ to look for possible polysialylated proteins in human serum. In this proteomic analysis, we observed not only NCAM as expected (Tajik et al. 2020), but also ST8Sia4 (Fig. 5C, Supplementary Table 2). ST8Sia4 has previously been detected in mass spectrometry analysis of serum (Dey et al. 2019) and given it autopolysialylates, it is not surprising it was observed in our analysis.

### QSOX2 is a novel polysialylated protein that is secreted from MCF-7 cells

**Fig. 6:**
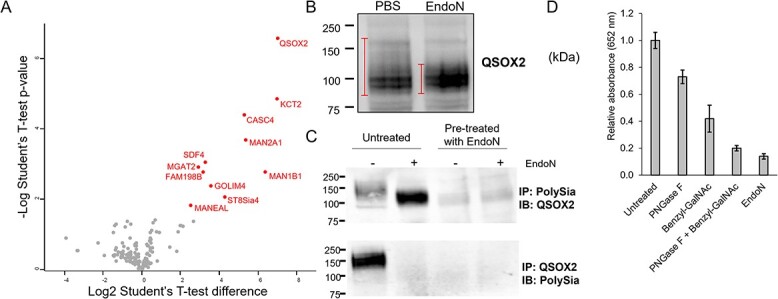
QSOX2 is a novel polysialylated protein. A) Volcano plot showing results from proteomic analysis of MCF-7 cell lysates (*n* = 4 per group, assessed with student’s T-test). The control lysates (left side) were pretreated with EndoN to hydrolyze polySia. B) Immunoblot of QSOX2 before and after treatment with EndoN. Bars represent areas with greater signal. C) Reciprocal immunoprecipitation of polysialylated QSOX2. Lysates were immunoprecipitated and then treated with PBS or EndoN before blotting (untreated +/− EndoN). As a negative control, lysates were also pretreated with EndoN before immunoprecipitation and then analyzed as before. D) PolySia-QSOX2 ELISA of MCF-7 cell lysates with PNGase F treatment to remove *N*-linked glycans, benzyl-GalNAc treatment to remove *O*-linked glycans, or both.

We next wanted to determine whether we could identify novel polysialylated proteins. MCF-7 cells are epithelial cells derived from a 69 year old woman with breast adenocarcinoma and have long been known to contain intracellular polySia that is not expressed on the cell surface (Martersteck et al. 1996). However, the identity of these polysialylated proteins has not yet been reported, though it is known that NCAM is not one (Fig. S8) (Martersteck et al. 1996). We isolated polysialylated proteins from MCF-7 cell lysates using immobilized EndoN_DM_, using EndoN-treated lysates as a negative control. Proteomic analysis revealed 11 putative polysialylated proteins (Fig. 6A, Supplementary Table 3). Of the 11, only the polysialyltransferase ST8Sia4 has previously been identified as being polysialylated. Most of the remaining proteins are normally associated with the Golgi. However, the most highly enriched protein was QSOX2, a member of the sulfhydryl oxidase/quiescin-6 (Q6) family of proteins that catalyze disulfide bond formation, which was recently found to be associated with poor outcomes in lung and colorectal cancers (Jiang et al. 2021; Li et al. 2021).

To validate the presence of polySia on QSOX2, we analyzed MCF-7 cell lysates by immunoblotting (Fig. 6B). We observed a reproducible smear slightly above two tight bands around 100 kDa. The predicted molecular weight of QSOX2 is 78 kDa so it is possible the bands at 100 kDa represent the glycosylated (but possibly non-polysialylated) form of QSOX2. Upon treatment of QSOX2 with EndoN, the higher molecular weight smear disappears and the band at 100 kDa becomes substantially brighter. To confirm that QSOX2 was indeed polysialylated, we performed reciprocal immunoprecipitation with α-QSOX2 and α-polySia antibodies (Fig. 6C, S9). After immunoprecipitating polySia, QSOX2 appeared as a higher molecular weight smear at ~150 kDa, which reduced to just over 100 kDa after treatment with EndoN. Pretreating the lysates with EndoN before immunoprecipitation abrogated any QSOX2 signal, confirming the specificity of the pull down. Similarly, immunoprecipitation of QSOX2 yielded an ~150 kDa band in polySia immunoblots, which disappeared after treatment with EndoN, providing unequivocal evidence that QSOX2 is polysialylated.

PolySia has been reported on both *N*-linked (NCAM) and *O*-linked (neuropilin-2) glycans. QSOX2 has 2 *N*-linked and at least 17 experimentally validated *O*-linked glycosylation sites (Steentoft et al. 2013). To determine whether *N*- or *O*-linked glycans are polysialylated on QSOX2 in MCF-7 cells, we analyzed cell lysates using a sandwich ELISA, where polysialylated proteins were isolated using the polySia lectin EndoN_DM_ adhered to 96 well plates and then the protein component was detected using an α-QSOX2 antibody (Tajik et al. 2020). Analysis of MCF-7 lysates yielded a robust signal for QSOX2 which was substantially reduced when pretreated with EndoN (Fig. 6D), again confirming the polysialylation of QSOX2. The linkage of glycans carrying polySia was determined using the *N*-glycosidase, PNGase F, and the *O*-linked glycosylation inhibitor, benzyl-GalNAc (Prescher and Bertozzi 2006; Wang and Voglmeir 2014). Treatment of cells with either benzyl-GalNAc or PNGase F both caused a decrease in signal for polySia-QSOX2 (Fig. 6D). Additionally, the combination of both treatments was equivalent to treatment with EndoN, indicating that both *N*- and *O*-linked glycans on QSOX2 are polysialylated.

**Fig. 7:**
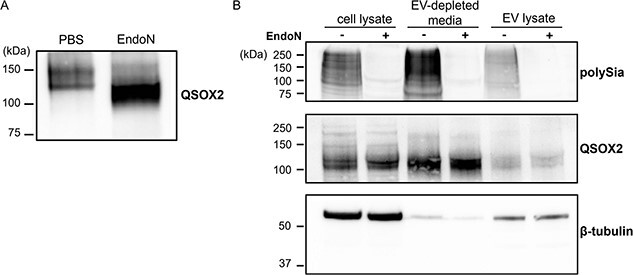
Polysialylated QSOX2 is secreted as a soluble protein from MCF-7 cells. A) α-QSOX2 immunoblot of polysialylated proteins immunoprecipitated from conditioned serum-free media after 3 days incubation. B) Immunoblot of MCF-7 cell lysate, extracellular vesicles (EV lysate), and conditioned media after removal of extracellular vesicles (EV-depleted media). Extracellular vesicles were isolated/removed by ultracentrifugation. Protein concentration of MCF-7 cell lysate, EV-depleted media, and EV lysate were determined using the BCA assay and an equivalent amount of protein was added in each well.

Given its similarity to QSOX1, we expected QSOX2 to be secreted from cells (Ilani et al. 2013) and predicted that the secreted form would be polysialylated. We immunoprecipitated polysialylated proteins from MCF-7 conditioned media and observed the high molecular weight QSOX2, which decreased in size after treatment with EndoN (Fig. 7A), confirming its secretion. To determine whether secreted QSOX2 was associated with extracellular vesicles (EVs), we analyzed EVs isolated by ultracentrifugation as well as EV-depleted media for the presence of QSOX2. The vast majority of QSOX2 remained associated with the EV-depleted media, suggesting that QSOX2 is secreted as a soluble protein (Fig. 7B).

## Discussion

EndoN and EndoN_DM_ have been important tools for polySia research since they were first characterized decades ago. Expanding on this original technology, we have developed new tools which will help investigate the mechanisms supporting polySia biology. We chose to use site specific immobilization of EndoN/EndoN_DM_ based on the 15 amino acid Avi-tag biotinylation sequence (Li and Sousa 2012). Previous experiments immobilizing EndoN_DM_ have relied on chemical crosslinking, like tosyl-activated beads (Kiermaier et al. 2016; Werneburg et al. 2016; Hsu et al. 2018). More recently, aldehyde cross-linking was also used to non-specifically immobilize a catalytically inactive point-mutant of the mucin protease StcE (Malaker et al. 2019). While chemical cross-linking is convenient, the heterogeneous nature of the immobilization may actually block the binding domains/active site in the immobilized protein. Additionally, if the protein of interest isn’t of high purity, non-specific proteins can also be immobilized, potentially complicating the analysis. By genetically encoding the Avi-tag into our protein of interest, we were able to efficiently biotinylate EndoN/EndoN_DM_ with recombinant BirA. The detergent-stability of EndoN/EndoN_DM_ and the near covalent interaction between streptavidin and biotin (Cronan Jr 1990; Stummeyer et al. 2005), allowed us to remove any non-specifically bound proteins after immobilization by washing with SDS, thereby providing an extra layer of specificity.

The immobilized EndoN will be useful when studying the interactions between cells expressing polySia or between these cells and their environment (e.g. serum and extracellular vesicles). Temporal experiments looking at polySia dynamics are also now possible with this methodology. While EndoN only removes cell surface polySia, combination experiments with the newly developed polySia biosynthesis inhibitor (Hunter et al. 2023) would allow for a more in depth exploration of polySia dynamics by targeting both cell surface and internal polySia.

Immobilized EndoN_DM_ represents an additional tool for isolating and identifying polysialylated proteins. Because the EndoN_DM_-biotin-streptavidin complex is detergent-stable, it is possible to perform the pull down in a high concentration of detergent to reduce non-specific interactions that frequently plague polySia-based pull downs. Additionally, the detergent can be removed through extensive washing, allowing for detergent-sensitive downstream applications, such as proteomics. Our proteomic analysis of NK-92 cells found that immobilized EndoN_DM_ significantly enriched NCAM and to a lesser extent ST8Sia4. This is one of the few polySia-proteomic experiments to identify a polysialyltransferase, despite the fact that both ST8Sia2 and ST8Sia4 autopolysialylate. We were also able to significantly enrich polysialylated proteins from serum using the immobilized EndoN_DM_, in contrast to mAb 735-based immunoprecipitation, which was plagued with high levels of non-specifically bound proteins. Similar to NK-92 cells, human serum contained both NCAM and ST8Sia4. NCAM/CD56 expressed on the surface of NK cells is susceptible to numerous proteases, including matrix metalloproteases MMP2/9 (Shichi et al. 2011), tumor necrosis factor-α-converting enzyme (TACE/ADAM17), and the β-site APP cleaving enzyme 1 (BACE1) secretase, so it may be released into serum after proteolysis. The mechanism by which ST8Sia4 gets into serum is less clear though it is also subject to proteolysis by BACE1 (Kitazume et al. 2006; Hemming et al. 2009). Alternatively, ST8Sia4 may be released into serum by activated platelets (Lee-Sundlov et al. 2017), and thus could have an impact on remodeling extracellular environments.

Using our immobilized EndoN_DM_, we also discovered that QSOX2 is a novel polysialylated protein. QSOX2 is a sulfhydryl oxidase which makes disulfides *de novo* (Reznik and Fass 2022). It is localized to the Golgi and is also secreted, consistent with our observations. QSOX2 is a relatively unstudied protein but is gaining attention because of its association with cancer. In a genetic study of colorectal cancer, QSOX2 overexpression was a predictor of poor prognosis (Nazempour et al. 2021). Knockdown of QSOX2 significantly reduced proliferation of colorectal cancer (Jiang et al. 2021) and non-small cell lung cancer cells (Li et al. 2021), and it was also identified as a potential serum biomarker for lung cancer (Li et al. 2021). Given the association of polySia with metastatic cancer, it will be interesting to determine how polysialylation affects QSOX2. While little is known about QSOX2 biology, the protein also shares 50% similarity with QSOX1, which is far better characterized (Reznik and Fass 2022). Like QSOX2, QSOX1 is a marker of metastasis in several cancers, including prostate (Ouyang et al. 2005; Baek et al. 2018), pancreatic (Antwi et al. 2009; Katchman et al. 2011), and breast (Katchman et al. 2013; Soloviev et al. 2013) cancers. Additionally, QSOX1/2 exclusively regulate disulfide bond formation in the Golgi (Reznik and Fass 2022). QSOX1 in particular is required for proper mucin production by goblet cells through regulation of ST3Gal1 and ST6Gal1 activity (Ilani et al. 2023). Whether QSOX2 is similarly important and whether polySia is required for this regulation remains to be determined. Secreted QSOX1 also remodels the extracellular matrix, one of the mechanisms by which it is connected to cancer metastasis (Ilani et al. 2013). It remains to be seen whether QSOX2 plays similar roles and whether polySia contributes directly to sulfhydryl oxidase activity or indirectly to QSOX2 biology by promoting ECM binding or increasing half-life (Lindhout et al. 2011).

In conclusion, the immobilized EndoN/EndoN_DM_ tools have the potential to improve the ability of researchers to design and interpret experiments investigating polySia biology, especially in areas of biology where polySia is less understood. Additionally, this method of site-specific immobilization can be used for immobilizing enzymes and lectins for studying other areas of glycobiology.

## Materials and methods

### Cells, antibodies, reagents

NK-92, K562, and MCF-7 cell lines were obtained from ATCC (CRL-2407, CCL-243, and HTB-22 respectively). NK-92 and K562 cells were cultured in RPMI-1640 (Gibco™, Cat# 11875093) supplemented with 10% horse serum (Gibco™, Cat# 16050122), 10% fetal bovine serum (Sigma-Aldrich), 25 mM HEPES pH 7, 100 U/mL penicillin-streptomycin (Gibco™, 10, 000 U/mL Cat# 15140-122), 200 U/mL IL-2 (Miltenyi Biotec), and 1 mM hydrocortisone (Stemcell). MCF-7 cells were cultured in Dulbecco’s Modified Eagle Medium (Cytiva SH30022.01) supplemented with 10% fetal bovine serum (Sigma-Aldrich) and 100 U/mL penicillin-streptomycin. Cells were incubated at 37 °C with 5% CO_2_ and media was changed every 2–3 days.

The antibodies used in this manuscript consisted of α-polySia mAb 735 (BioAspect BA-Ab00240-2.0), α-CD56 (R&D systems, mAB2408), α-beta-tubulin (Abcam, ab6046), streptavidin-HRP (Biolegend, 405210), goat α-mouse IgG-HRP (R&D systems, HAF007), goat α-rabbit IgG-HRP (Abcam, ab6721), and α-QSOX2 (Abcam, ab191168 and ab121376).

### Cloning

The His_6_-Avi-EndoN_DM_ was constructed from a 3-way ligation. The plasmid expressing GFP-EndoN_DM_ (Yu et al. 2013) was digested with *BamHI*-HF and *NheI* restriction enzymes to produce the *NheI-BamHI* vector fragment and the *BamHI-Bam-HI* EndoN_DM_ insert. Primers corresponding to *NheI-BamHI* Avi tag were phosphorylated with the T4 polynucleotide kinase (NEB) and annealed. Equimolar amounts of the Avi tag and EndoN_DM_ fragments were ligated for 1 h before adding the vector fragment and allowing the ligation to proceed overnight. *E. coli* BL21 (DE3) competent cells were transformed with the ligation reaction and positive transformants were verified by sequencing. To produce the His_6_-Avi-EndoN construct, the gene encoding the active EndoN was subcloned from pCWmalEendoN (Morley et al. 2009) into the newly made His_6_-Avi-EndoN_DM_ construct using *AgeI* and *SalI/XhoI* sites. This plasmid was also verified by sequencing.

### Protein expression and purification

Verified transformants were cultured overnight in Luria Broth (LB) media at 37 °C. The overnight cultures were diluted 1/100 in LB media supplemented with 100 μg/mL ampicillin. Cultures were grown at 37 °C with shaking for 2 h. To induce the recombinant protein expression, 0.5 mM isopropyl-thio-β-galactoside (IPTG) was added to the cultures and cells were incubated at 20 °C for 26 h with shaking. Cells were harvested and lysed in buffer containing 20 mM Tris-HCl (pH 8), 500 mM NaCl, 10 mM β-mercaptoethanol, 10% glycerol, and 10 mM imidazole, protease inhibitor cocktail (Roche), and benzonase (EMD Millipore). The supernatant was cleared by 30 min centrifugation at 15,000 × *g* at 4 °C. The supernatant was passed through the cOmplete His-Tag purification resin (Roche) column and recombinant proteins were eluted in 20 mM Tris-HCl (pH 8), 500 mM NaCl, 10 mM β- mercaptoethanol, 10% glycerol, 300 mM imidazole. The purity of the fractions was assessed by SDS-PAGE. The fractions were pooled, and final protein concentration was measured by BCA assay.

MalE-EndoN was expressed and purified as previously described (Morley et al. 2009).

BirA was expressed and purified as previously described (Li and Sousa 2012).

### Conjugation of EndoN to streptavidin-coated agarose or magnetic beads

1 mg of purified His_6_-Avi-EndoN or His_6_-Avi-EndoN_DM_ were biotinylated as previously described (Li and Sousa 2012). Briefly, Avi-tagged proteins and BirA were incubated together with 1:0.01 ratio in the presence of 0.3 mM biotin and 5 mM ATP in 25 mM Tris-HCl pH 8 for 4 h at room temperature with rotating. Excess biotin was removed from the reaction and protein was concentrated by filtering though 3 K MWCO Amicon centrifugal units to a final concentration of 0.5 mg/mL protein in PBS. Biotinylation was assessed by analysis of 0.25 μg protein by SDS-PAGE and blotting with HRP-conjugated streptavidin. The blot was developed using an ECL chemiluminescence substrate kit (Thermo Scientific).

A streptavidin-agarose slurry (Sigma) was washed with PBS prior to adding biotinylated His_6_-Avi-EndoN or His_6_-Avi-EndoN_DM_. The protein was incubated with the beads at room temperature for 1 h with rotation. Following incubation, beads were transferred to mini columns (Thermo Scientific) for washing. They were washed with 0.1% BSA in PBS-Tween and then PBS, before storing in PBS at 4 °C.

To conjugate biotinylated His_6-_Avi-Endo-N to magnetic streptavidin beads (New England Biolabs), the beads were washed with PBS and incubated with biotinylated EndoN for 1 h at room temperature with rotation. For removing unbound enzyme and other contaminants, the complex was washed three times with 1% SDS in PBS and then seven times with PBS.

### Mixing experiments

Pre-treatment phase: A total of 10^6^ NK-92 cells were collected and washed three times with PBS. The cells were treated with either 5 μg/mL soluble EndoN, roughly 5 μg/mL immobilized EndoN (assuming 100% efficiency of adhering to streptavidin beads), or PBS for 0.5 h at 37 °C. To remove the soluble enzyme, EndoN treated cells were collected by centrifugation at 180 × *g* and washed three times with 1 mL PBS. To remove immobilized EndoN, cells were incubated in a magnet (EasySep™ Magnet, STEMCELL Technologies) for 5 min, allowing the magnetic beads to adhere to the sides of the tubes and the cells to be collected in a fresh tube. For K562 cells, 10^6^ cells were incubated in PBS for 0.5 h at 37 °C, then washed three times with PBS for consistency.

Mixing phase: Cells from two treatment conditions were mixed together and incubated at 37 °C for 1 h. The mixed cells were then either lysed for immunoblot analysis or prepared for flow cytometry analysis.

### Immunoblotting

Cells were lysed with RIPA buffer containing 50 mM Tris-HCl pH 8, 150 mM NaCl, 0.1% SDS, 0.5% sodium deoxycholate, 1% NP-40, 1 mM phenylmethylsulfonyl fluoride (PMSF), and benzonase (EMD Millipore) for 30 min on ice, followed by centrifugation for 10 min at 10,000 × *g*. Protein concentration was determined using the BCA protein assay. 7–10 μg protein from NK-92 cell lysates or 8–30 μg protein from MCF-7 cell lysates were resolved by SDS-PAGE and transferred to a PVDF membrane that was then blocked with 5% bovine serum albumin (BSA). The membrane was incubated with α-polySia (1:1000), α-CD56 (1:500), or rabbit α-beta-tubulin (1:2000) in PBS-Tween or α-QSOX-2 (1:500) in 5% BSA in PBS-Tween overnight at room temperature. The membrane was washed three times with PBS-Tween and incubated for one hour with 1:1000 α-mouse or α-rabbit IgG conjugated to HRP. The membrane was washed three times with PBS-Tween followed by development using Pierce ECL Western Blotting Substrate (Thermo Fisher).

For analysis of biotinylated proteins, the blot was incubated with 1:5000 streptavidin-HRP in 5% BSA, 1X PBS-Tween for 1 h. The membrane was washed three times with PBS-Tween followed by development using Pierce ECL Western Blotting Substrate.

### Flow cytometry

For flow cytometry analysis, cells were incubated with Zombie NIR flexible viability kit (Biolegend) with 1:200 dilution for 20 min to label dead cells. The FC receptors were blocked with Human TruStain FCXTM (Biolegend) for 20 min, followed by 30 min of incubation with anti-CD56 (1:100 dilution) and 4 μg/mL GFP-EndoN_DM_. Cells were washed and fixed with 1% formaldehyde (Thermo Scientific), then fluorescence signal was acquired on the Cytek Aurora and analyzed using FlowJo V10.8.1.

### Sample preparation for proteomic analysis

Cells were harvested three days after seeding by centrifugation at 360 × *g* and washed three times with PBS. Cells were lysed in RIPA buffer as described above. Cell lysate was either treated with EndoN or PBS and then added to 100 μL of biotinylated EndoN_DM_ bead slurry and 50 mM sodium phosphate (Na_x_H_x_PO_4_), 130 mM NaCl pH 8. This mixture was incubated for 1 h at room temperature with rotation followed by separation of beads from the flowthrough using mini columns (Thermo Scientific). The beads were then washed with 2.5 mL of 50 mM Na_x_H_x_PO_4_, 1.5 M NaCl, pH 8, followed by 2.5 mL 1% SDS in PBS, 5 mL PBS, and finally 1 mL 50 mM NH_4_HCO_4_. Beads were transferred to a new tube with 50 mM NH_4_HCO_4,_ and liquid was removed before proteomic analysis. Serum (500 μL) was diluted with an equivalent volume of PBS. SDS was added to 1% and 35 μL of biotinylated EndoN_DM_ bead slurry was added. The mixture was incubated for 1.5 h at room temperature with rotation. The beads were transferred to mini columns and washed as described above. For immunoblotting samples at this stage were mixed with loading dye and heated for 3 min at 95 °C before being run on SDS-PAGE gels.

Beads containing the polySia enriched proteins or mock enrichment controls were solubilized in 4% SDS, 100 mM Tris pH 8.5 by boiling for 10 min at 95 °C. The resulting lysate was prepared for digestion using Micro S-traps (Protifi, USA) according to the manufacturer’s instructions. Briefly, samples were reduced with 10 mM DTT for 10 min at 95 °C and then alkylated with 40 mM IAA in the dark for 1 h. Reduced and alkylated samples, including the beads, were acidified to 1.2% phosphoric acid then diluted with seven volumes of S-trap wash buffer (90% methanol, 100 mM Tetraethylammonium bromide pH 7.1) before being loaded onto the S-traps and washed 3 times with S-trap wash buffer. Samples were then digested with 2 μg of Trypsin overnight before being collected by centrifugation with washes of 100 mM tetraethylammonium bromide, followed by 0.2% formic acid followed by 0.2% formic acid / 50% acetonitrile. Samples were dried down and further cleaned up using C18 Stage (Rappsilber et al. 2003; Rappsilber et al. 2007) tips to ensure the removal of any particulate matter.

### Reverse phase liquid chromatography–mass spectrometry

Prepared purified peptides from each sample were re-suspended in Buffer A* (2% acetonitrile, 0.01% trifluoroacetic acid) and separated using a two-column chromatography setup composed of a PepMap100 C_18_ 20-mm by 75-μm trap and a PepMap C_18_ 500-mm by 75-μm analytical column (Thermo Fisher Scientific). Samples were concentrated onto the trap column at 5 μL/min for 5 min with Buffer A (0.1% formic acid, 2% DMSO) and then infused into an Orbitrap Fusion Lumos or Orbitrap Q-exactive plus (Thermo Fisher Scientific) at 300 nL/min via the analytical columns using a Dionex Ultimate 3,000 UPLC (Thermo Fisher Scientific). Samples were separated using a 95-min analytical gradient undertaken by altering the buffer composition from 2% Buffer B (0.1% formic acid, 77.9% acetonitrile, 2% DMSO) to 22% B over 65 min, then from 22% B to 40% B over 10 min, then from 40% B to 80% B over 5 min. The composition was held at 80% B for 5 min, and then dropped to 2% B over 2 min before being held at 2% B for another 8 min. The Orbitrap Lumos mass spectrometer was operated in a data-dependent mode automatically switching between the acquisition of a single Orbitrap MS scan (300–1,500 m/z and a resolution of 120 k) and 3 s of Orbitrap MS/MS HCD scans of precursors (Stepped NCE of 35;30 and 35%, a maximal injection time of 80 ms, an Automatic Gain Control (AGC) value of 250% and a resolution of 30 k). The Orbitrap Q-exactive plust mass spectrometer was operated in a data-dependent mode automatically switching between the acquisition of a single Orbitrap MS scan (375–1,800 m/z and a resolution of 120 k) and 15 Orbitrap MS/MS HCD scans of precursors (Stepped NCE of 28;30 and 25%, a maximal injection time of 110 ms, an Automatic Gain Control (AGC) value of 2e5 and a resolution of 35 k).

### Proteomic data analysis

Identification and LFQ analysis were accomplished using MaxQuant (v1.6.17.0) (Cox and Mann 2008). Data was searched against the human proteome (Uniprot: UP000005640) allowing for oxidation on Methionine. The LFQ and “Match Between Run” options were enabled to allow comparison between samples. The resulting data files were processed using Perseus (v1.4.0.6) (Tyanova et al. 2016) to compare the enrichment of proteins using student T-tests. The mass spectrometry proteomics data have been deposited to the ProteomeXchange Consortium via the PRIDE (Perez-Riverol et al. 2022) partner repository with the dataset identifier PXD040100 (Reviewer account details: Username: reviewer_pxd040100@ebi.ac.uk Password: 4XiaWA7A) or or PXD046194 (Reviewer account details: Username: reviewer_pxd046194@ebi.ac.uk Password: o2U1bq8Q).

### Determination of polySia glycan linkages

MCF-7 cells at ~40% confluency were treated with the *O*-linked glycosylation inhibitor, benzyl 2-acetamido-2-deoxy-α-D-galactopyranoside (benzyl-GalNAc; Sigma), at 2 mM for three days. Lysates from cells +/− benzyl-GalNAc were prepared as for immunoblotting. 1,200 μg protein was mixed with 15 μL Glycoprotein Denaturing Buffer (NEB) in 150 μL total volume and incubated at room temperature for 10 min. 30 μL 10% NP-40, 30 μL Glyco Buffer 2, 90 μL dH_2_O and +/− 2,500 U PNGase F (NEB) was added to each tube and incubated at 37 °C for 1.5 h. Samples were analysed directly by ELISA (Tajik et al. 2020). Error bars denote standard deviations on biological replicates run at least three times in technical duplicate.

### Reciprocal immunoprecipitation

MCF-7 cells were lysed in 0.1% Triton in PBS containing benzonase and 1 mM PMSF. 80 μg of cell lysate in 100 μL 0.1% Triton in PBS was treated with 7 μg of EndoN or an equivalent volume of PBS for 30 min at 37 °C. Cell lysate was incubated with 2 μg α-polySia mAb 735 or 0.5 μg α-QSOX2 (abcam 121376) overnight at 4 °C with rotation. 5 μL of protein G beads washed in 0.1% Triton in PBS were added to each sample and the mixture was incubated for 4 h at 4 °C with rotation. The beads were washed 3 times in 0.2% Triton in PBS and samples were eluted with SDS-PAGE loading dye through heating for 3 min at 95 °C.

### Comparison of polySia pulldown with EndoN_DM_ vs immunoprecipitation with mAb735

250 μL of healthy human serum was used for each method. EndoN_DM_ pulldown and mAb735 immunoprecipitations were performed as described, and beads were resuspended in equivalent volumes for analysis. For no serum controls, an equivalent volume of PBS was used in place of serum.

### Analysis of secreted polysialylated QSOX2

MCF-7 cells at ~40% confluency were washed with PBS then incubated with Opti-MEM (Gibco) supplemented with 30 mM glucose, 2 mM CaCl_2_, 40 mM non-essential amino acids (Thermo Fisher), 10 ng/mL hydrocortisone, and 1X penicillin–streptomycin at 37 °C for three days. Media was collected and floating cells were removed by centrifugation at 300 × g for 10 min. The supernatant was spun at 2,000 × g for 10 min to remove additional debris. EVs were pelleted by ultracentrifugation at 100,000 × g for 70 min at 2 °C. The EV-depleted media (supernatant) was retained and concentrated for analysis. The pellet (EVs) were resuspended in PBS and recentrifuged at 100,000 × g for 70 min at 2 °C, then resuspended in 100 μL PBS.

## Author contributions

Carmanah D. Hunter (Conceptualization [equal], Data curation [equal], Formal analysis [equal], Investigation [equal], Methodology [equal], Supervision [equal], Validation [equal], Writing—original draft [equal], Writing—review & editing [equal]), Tahlia Derksen (Conceptualization [supporting], Data curation [equal], Formal analysis [equal], Investigation [equal], Methodology [equal], Visualization [equal], Writing—original draft [equal], Writing—review & editing [equal]), Sogand Makhsous (Conceptualization [supporting], Data curation [equal], Formal analysis [equal], Investigation [equal], Methodology [equal], Visualization [equal], Writing—original draft [equal], Writing—review & editing [equal]), Matt Doll (Investigation [equal], Methodology [equal], Writing—review & editing [equal]), Samantha Rodriguez Perez (Investigation [equal], Methodology [equal]), Nichollas Scott (Conceptualization [equal], Funding acquisition [equal], Investigation [equal], Methodology [equal], Writing—review & editing [equal]), and Lisa Willis (Conceptualization [equal], Formal analysis [equal], Funding acquisition [equal], Investigation [equal], Methodology [equal], Supervision [equal], Writing—original draft [equal], Writing—review & editing [equal])

## Supplementary Material

Supplemental_v4_cwae026

Supplementary_table_I_proteomic_results_cwae026

Supplementary_table_II_proteomic_results_cwae026

Supplementary_table_III_proteomic_results_cwae026

## Data Availability

Source data are provided with this paper.

## References

[ref1] Adams OJ , Stanczak MA, von Gunten S, Laubli H. Targeting sialic acid-Siglec interactions to reverse immune suppression in cancer. Glycobiology. 2018:28(9):640–647. 29309569 10.1093/glycob/cwx108

[ref2] Anney R , Klei L, Pinto D, Regan R, Conroy J, Magalhaes TR, Correia C, Abrahams BS, Sykes N, Pagnamenta AT, et al. A genome-wide scan for common alleles affecting risk for autism. Hum Mol Genet. 2010:19(20):4072–4082. 20663923 10.1093/hmg/ddq307 PMC2947401

[ref3] Antwi K , Hostetter G, Demeure MJ, Katchman BA, Decker GA, Ruiz Y, Sielaff TD, Koep LJ, Lake DF. Analysis of the plasma peptidome from pancreas cancer patients connects a peptide in plasma to overexpression of the parent protein in tumors. J Proteome Res. 2009:8(10):4722–4731. 19795908 10.1021/pr900414f

[ref4] Baek JA , Song PH, Ko Y, Gu MJ. High expression of QSOX1 is associated with tumor invasiveness and high grades groups in prostate cancer. Pathol Res Pract. 2018:214(7):964–967. 29804717 10.1016/j.prp.2018.05.019

[ref5] Barenwaldt A , Laubli H. The sialoglycan-Siglec glyco-immune checkpoint - a target for improving innate and adaptive anti-cancer immunity. Expert Opin Ther Targets. 2019:23(10):839–853. 31524529 10.1080/14728222.2019.1667977

[ref6] Colley KJ , Kitajima K, Sato C. Polysialic acid: biosynthesis, novel functions and applications. Crit Rev Biochem Mol Biol. 2014:49(6):498–532. 25373518 10.3109/10409238.2014.976606

[ref7] Cox J , Mann M. MaxQuant enables high peptide identification rates, individualized p.p.b.-range mass accuracies and proteome-wide protein quantification. Nat Biotechnol. 2008:26(12):1367–1372. 19029910 10.1038/nbt.1511

[ref8] Cronan JE Jr . Biotination of proteins in vivo. A post-translational modification to label, purify, and study proteins. J Biol Chem. 1990:265(18):10327–10333. 2113052

[ref9] Curreli S , Arany Z, Gerardy-Schahn R, Mann D, Stamatos NM. Polysialylated neuropilin-2 is expressed on the surface of human dendritic cells and modulates dendritic cell-T lymphocyte interactions. J Biol Chem. 2007:282(42):30346–30356. 17699524 10.1074/jbc.M702965200

[ref10] Daniel L , Durbec P, Gautherot E, Rouvier E, Rougon G, Figarella-Branger D. A nude mice model of human rhabdomyosarcoma lung metastases for evaluating the role of polysialic acids in the metastatic process. Oncogene. 2001:20(8):997–1004. 11314035 10.1038/sj.onc.1204176

[ref11] Dey KK , Wang H, Niu M, Bai B, Wang X, Li Y, Cho J-H, Tan H, Mishra A, High AA, et al. Deep undepleted human serum proteome profiling toward biomarker discovery for Alzheimer’s disease. Clin Proteomics. 2019:16(1):16. 31019427 10.1186/s12014-019-9237-1 PMC6472024

[ref12] El Maarouf A , Rutishauser U. Removal of polysialic acid induces aberrant pathways, synaptic vesicle distribution, and terminal arborization of retinotectal axons. J Comp Neurol. 2003:460(2):203–211. 12687685 10.1002/cne.10635

[ref13] Falconer RA , Errington RJ, Shnyder SD, Smith PJ, Patterson LH. Polysialyltransferase: a new target in metastatic cancer. Curr Cancer Drug Targets. 2012:12(8):925–939. 22463390 10.2174/156800912803251225

[ref14] Franceschini I , Angata K, Ong E, Hong A, Doherty P, Fukuda M. Polysialyltransferase ST8Sia II (STX) polysialylates all of the major isoforms of NCAM and facilitates neurite outgrowth. Glycobiology. 2001:11(3):231–239. 11320061 10.1093/glycob/11.3.231

[ref15] Fullerton JM , Klauser P, Lenroot RK, Shaw AD, Overs B, Heath A, Cairns MJ, Atkins J, Scott R, Schofield PR, et al. Differential effect of disease-associated ST8SIA2 haplotype on cerebral white matter diffusion properties in schizophrenia and healthy controls. Transl Psychiatry. 2018:8(1):21. 29353880 10.1038/s41398-017-0052-z PMC5802561

[ref16] Gong L , Zhou X, Yang J, Jiang Y, Yang H. Effects of the regulation of polysialyltransferase ST8SiaII on the invasiveness and metastasis of small cell lung cancer cells. Oncol Rep. 2017:37(1):131–138. 28004110 10.3892/or.2016.5279

[ref17] Häyrinen J , Haseley S, Talaga P, Mühlenhoff M, Finne J, Vliegenthart JF. High affinity binding of long-chain polysialic acid to antibody, and modulation by divalent cations and polyamines. Mol Immunol. 2002:39(7-8):399–411. 12413691 10.1016/s0161-5890(02)00202-x

[ref18] Heimburg-Molinaro J , Lum M, Vijay G, Jain M, Almogren A, Rittenhouse-Olson K. Cancer vaccines and carbohydrate epitopes. Vaccine. 2011:29(48):8802–8826. 21964054 10.1016/j.vaccine.2011.09.009 PMC3208265

[ref19] Hemming ML , Elias JE, Gygi SP, Selkoe DJ. Identification of beta-secretase (BACE1) substrates using quantitative proteomics. PLoS One. 2009:4(12):e8477. 20041192 10.1371/journal.pone.0008477 PMC2793532

[ref20] Hsu H-J , Palka-Hamblin H, Bhide GP, Myung J-H, Cheong M, Colley KJ, Hong S. Noncatalytic endosialidase enables surface capture of small-cell lung cancer cells utilizing strong dendrimer-mediated enzyme-glycoprotein interactions. Anal Chem. 2018:90(6):3670–3675. 29473730 10.1021/acs.analchem.8b00427 PMC7038578

[ref21] Hunter C , Gao Z, Chen HM, Thompson N, Wakarchuk W, Nitz M, Withers SG, Willis LM. Attenuation of polysialic acid biosynthesis in cells by the small molecule inhibitor 8-Keto-sialic acid. ACS Chem Biol. 2023:18(1):41–48. 36577399 10.1021/acschembio.2c00638

[ref22] Ilani T , Alon A, Grossman I, Horowitz B, Kartvelishvily E, Cohen SR, Fass D. A secreted disulfide catalyst controls extracellular matrix composition and function. Science. 2013:341(6141):74–76. 23704371 10.1126/science.1238279

[ref23] Ilani T , Reznik N, Yeshaya N, Feldman T, Vilela P, Lansky Z, Javitt G, Shemesh M, Brenner O, Elkis Y, et al. The disulfide catalyst QSOX1 maintains the colon mucosal barrier by regulating Golgi glycosyltransferases. EMBO J. 2023:42(2):e111869. 36245281 10.15252/embj.2022111869 PMC9841341

[ref24] Jazayeri MH , Pourfathollah AA, Rasaee MJ, Porpak Z, Jafari ME. The concentration of total serum IgG and IgM in sera of healthy individuals varies at different age intervals. Biomed Aging Pathol. 2013:3(4):241–245.

[ref25] Jiang T , Zheng L, Li X, Liu J, Song H, Xu Y, Dong C, Liu L, Wang H, Wang S, et al. Quiescin sulfhydryl oxidase 2 overexpression predicts poor prognosis and tumor progression in patients with colorectal cancer: a study based on data mining and clinical verification. Front Cell Dev Biol. 2021:9:678770. 34858968 10.3389/fcell.2021.678770 PMC8631333

[ref26] Jokilammi A , Ollikka P, Korja M, Jakobsson E, Loimaranta V, Haataja S, Hirvonen H, Finne J. Construction of antibody mimics from a noncatalytic enzyme-detection of polysialic acid. J Immunol Methods. 2004:295(1-2):149–160. 15627620 10.1016/j.jim.2004.10.006

[ref27] Karlstetter M , Kopatz J, Aslanidis A, Shahraz A, Caramoy A, Linnartz-Gerlach B, Lin Y, Lückoff A, Fauser S, Düker K, et al. Polysialic acid blocks mononuclear phagocyte reactivity, inhibits complement activation, and protects from vascular damage in the retina. EMBO Mol Med. 2017:9(2):154–166. 28003336 10.15252/emmm.201606627 PMC5286381

[ref28] Katchman BA , Antwi K, Hostetter G, Demeure MJ, Watanabe A, Decker GA, Miller LJ, Von Hoff DD, Lake DF. Quiescin sulfhydryl oxidase 1 promotes invasion of pancreatic tumor cells mediated by matrix metalloproteinases. Mol Cancer Res. 2011:9(12):1621–1631. 21989104 10.1158/1541-7786.MCR-11-0018

[ref29] Katchman BA , Ocal IT, Cunliffe HE, Chang YH, Hostetter G, Watanabe A, LoBello J, Lake DF. Expression of quiescin sulfhydryl oxidase 1 is associated with a highly invasive phenotype and correlates with a poor prognosis in luminal B breast cancer. Breast Cancer Res. 2013:15(2):R28. 23536962 10.1186/bcr3407 PMC3738157

[ref30] Khan L , Derksen T, Redmond D, Storek J, Durand C, Gniadecki R, Korman B, Cohen Tervaert JW, D'Aubeterre A, Osman MS, et al. The cancer-associated glycan polysialic acid is dysregulated in systemic sclerosis and is associated with fibrosis. J Autoimmun. 2023:140:103110. 37742510 10.1016/j.jaut.2023.103110

[ref31] Kiermaier E , Moussion C, Veldkamp CT, Gerardy-Schahn R, de Vries I, Williams LG, Chaffee GR, Phillips AJ, Freiberger F, Imre R, et al. Polysialylation controls dendritic cell trafficking by regulating chemokine recognition. Science. 2016:351(6269):186–190. 26657283 10.1126/science.aad0512 PMC5583642

[ref32] Kitazume S , Tachida Y, Oka R, Nakagawa K, Takashima S, Lee YC, Hashimoto Y. Screening a series of sialyltransferases for possible BACE1 substrates. Glycoconj J. 2006:23(5-6):437–441. 16897184 10.1007/s10719-006-6671-x

[ref33] Lee-Sundlov MM , Ashline DJ, Hanneman AJ, Grozovsky R, Reinhold VN, Hoffmeister KM, Lau JT. Circulating blood and platelets supply glycosyltransferases that enable extrinsic extracellular glycosylation. Glycobiology. 2017:27(2):188–198. 27798070 10.1093/glycob/cww108 PMC5224594

[ref34] Li Y , Sousa R. Expression and purification of E. Coli BirA biotin ligase for in vitro biotinylation. Protein Expr Purif. 2012:82(1):162–167. 22227598 10.1016/j.pep.2011.12.008 PMC3288220

[ref35] Li Y , Liu M, Zhang Z, Deng L, Zhai Z, Liu H, Wang Y, Zhang C, Xiong J, Shi C. QSOX2 is an E2F1 target gene and a novel serum biomarker for monitoring tumor growth and predicting survival in advanced NSCLC. Front Cell Dev Biol. 2021:9:688798. 34350181 10.3389/fcell.2021.688798 PMC8326667

[ref36] Lindhout T , Iqbal U, Willis LM, Reid AN, Li J, Liu X, Moreno M, Wakarchuk WW. Site-specific enzymatic polysialylation of therapeutic proteins using bacterial enzymes. Proc Natl Acad Sci USA. 2011:108(18):7397–7402. 21502532 10.1073/pnas.1019266108 PMC3088639

[ref37] Maheu ME , Davoli MA, Turecki G, Mechawar N. Amygdalar expression of proteins associated with neuroplasticity in major depression and suicide. J Psychiatr Res. 2013:47(3):384–390. 23260340 10.1016/j.jpsychires.2012.11.013

[ref38] Malaker SA , Pedram K, Ferracane MJ, Bensing BA, Krishnan V, Pett C, Yu J, Woods EC, Kramer JR, Westerlind U, et al. The mucin-selective protease StcE enables molecular and functional analysis of human cancer-associated mucins. Proc Natl Acad Sci USA. 2019:116(15):7278–7287. 30910957 10.1073/pnas.1813020116 PMC6462054

[ref39] Martersteck CM , Kedersha NL, Drapp DA, Tsui TG, Colley KJ. Unique alpha 2, 8-polysialylated glycoproteins in breast cancer and leukemia cells. Glycobiology. 1996:6(3):289–301. 8724137 10.1093/glycob/6.3.289

[ref40] McAuley EZ , Blair IP, Liu Z, Fullerton JM, Scimone A, Van Herten M, Evans MR, Kirkby KC, Donald JA, Mitchell PB, et al. A genome screen of 35 bipolar affective disorder pedigrees provides significant evidence for a susceptibility locus on chromosome 15q25-26. Mol Psychiatry. 2009:14(5):492–500. 18227837 10.1038/sj.mp.4002146

[ref41] Mikkonen M , Soininen H, Tapiola T, Alafuzoff I, Miettinen R. Hippocampal plasticity in Alzheimer's disease: changes in highly polysialylated NCAM immunoreactivity in the hippocampal formation. Eur J Neurosci. 1999:11(5):1754–1764. 10215928 10.1046/j.1460-9568.1999.00593.x

[ref42] Morley TJ , Willis LM, Whitfield C, Wakarchuk WW, Withers SG. A new sialidase mechanism: bacteriophage K1F endo-sialidase is an inverting glycosidase. J Biol Chem. 2009:284(26):17404–17410. 19411257 10.1074/jbc.M109.003970 PMC2719380

[ref43] Nakata D , Troy FA 2nd. Degree of polymerization (DP) of polysialic acid (polySia) on neural cell adhesion molecules (N-CAMS): development and application of a new strategy to accurately determine the DP of polySia chains on N-CAMS. J Biol Chem. 2005:280(46):38305–38316. 16172115 10.1074/jbc.M508762200

[ref44] Nazempour N , Taleqani MH, Taheri N, Haji Ali Asgary Najafabadi AH, Shokrollahi A, Zamani A, Fattahi Dolatabadi N, Peymani M, Mahdevar M. The role of cell surface proteins gene expression in diagnosis, prognosis, and drug resistance of colorectal cancer: in silico analysis and validation. Exp Mol Pathol. 2021:123:104688. 34592197 10.1016/j.yexmp.2021.104688

[ref45] Ouyang X , DeWeese TL, Nelson WG, Abate-Shen C. Loss-of-function of Nkx3.1 promotes increased oxidative damage in prostate carcinogenesis. Cancer Res. 2005:65(15):6773–6779. 16061659 10.1158/0008-5472.CAN-05-1948

[ref46] Pelkonen S , Aalto J, Finne J. Differential activities of bacteriophage depolymerase on bacterial polysaccharide: binding is essential but degradation is inhibitory in phage infection of K1-defective Escherichia coli. J Bacteriol. 1992:174(23):7757–7761. 1447142 10.1128/jb.174.23.7757-7761.1992 PMC207490

[ref47] Perez-Riverol Y , Bai J, Bandla C, García-Seisdedos D, Hewapathirana S, Kamatchinathan S, Kundu DJ, Prakash A, Frericks-Zipper A, Eisenacher M, et al. The PRIDE database resources in 2022: a hub for mass spectrometry-based proteomics evidences. Nucleic Acids Res. 2022:50(D1):D543–d552. 34723319 10.1093/nar/gkab1038 PMC8728295

[ref48] Piras F , Schiff M, Chiapponi C, Bossu P, Muhlenhoff M, Caltagirone C, Gerardy-Schahn R, Hildebrandt H, Spalletta G. Brain structure, cognition and negative symptoms in schizophrenia are associated with serum levels of polysialic acid-modified NCAM. Transl Psychiatry. 2015:5(10):e658. 26460482 10.1038/tp.2015.156 PMC4930132

[ref49] Prescher JA , Bertozzi CR. Chemical technologies for probing glycans. Cell. 2006:126(5):851–854. 16959565 10.1016/j.cell.2006.08.017

[ref50] Rappsilber J , Ishihama Y, Mann M. Stop and go extraction tips for matrix-assisted laser desorption/ionization, nanoelectrospray, and LC/MS sample pretreatment in proteomics. Anal Chem. 2003:75(3):663–670. 12585499 10.1021/ac026117i

[ref51] Rappsilber J , Mann M, Ishihama Y. Protocol for micro-purification, enrichment, pre-fractionation and storage of peptides for proteomics using StageTips. Nat Protoc. 2007:2(8):1896–1906. 17703201 10.1038/nprot.2007.261

[ref52] Rey-Gallardo A , Escribano C, Delgado-Martin C, Rodriguez-Fernandez JL, Gerardy-Schahn R, Rutishauser U, Corbi AL, Vega MA. Polysialylated neuropilin-2 enhances human dendritic cell migration through the basic C-terminal region of CCL21. Glycobiology. 2010:20(9):1139–1146. 20488940 10.1093/glycob/cwq078

[ref53] Reznik N , Fass D. Disulfide bond formation and redox regulation in the Golgi apparatus. FEBS Lett. 2022:596(22):2859–2872. 36214053 10.1002/1873-3468.14510

[ref54] Schnaar RL , Gerardy-Schahn R, Hildebrandt H. Sialic acids in the brain: gangliosides and polysialic acid in nervous system development, stability, disease, and regeneration. Physiol Rev. 2014:94(2):461–518. 24692354 10.1152/physrev.00033.2013 PMC4044301

[ref55] Shichi K , Fujita-Hamabe W, Harada S, Mizoguchi H, Yamada K, Nabeshima T, Tokuyama S. Involvement of matrix metalloproteinase-mediated proteolysis of neural cell adhesion molecule in the development of cerebral ischemic neuronal damage. J Pharmacol Exp Ther. 2011:338(2):701–710. 21602423 10.1124/jpet.110.178079

[ref56] Simon P , Baumner S, Busch O, Rohrich R, Kaese M, Richterich P, Wehrend A, Muller K, Gerardy-Schahn R, Muhlenhoff M, et al. Polysialic acid is present in mammalian semen as a post-translational modification of the neural cell adhesion molecule NCAM and the polysialyltransferase ST8SiaII. J Biol Chem. 2013:288(26):18825–18833. 23671285 10.1074/jbc.M113.451112 PMC3696658

[ref57] Soloviev M , Esteves MP, Amiri F, Crompton MR, Rider CC. Elevated transcription of the gene QSOX1 encoding quiescin Q6 sulfhydryl oxidase 1 in breast cancer. PLoS One. 2013:8(2):e57327. 23460839 10.1371/journal.pone.0057327 PMC3583868

[ref58] Stamatos NM , Zhang L, Jokilammi A, Finne J, Chen WH, El-Maarouf A, Cross AS, Hankey KG. Changes in polysialic acid expression on myeloid cells during differentiation and recruitment to sites of inflammation: role in phagocytosis. Glycobiology. 2014:24(9):864–879. 24865221 10.1093/glycob/cwu050 PMC4116047

[ref59] Steentoft C , Vakhrushev SY, Joshi HJ, Kong Y, Vester-Christensen MB, Schjoldager KT-BG, Lavrsen K, Dabelsteen S, Pedersen NB, Marcos-Silva L, et al. Precision mapping of the human O-GalNAc glycoproteome through SimpleCell technology. EMBO J. 2013:32(10):1478–1488. 23584533 10.1038/emboj.2013.79 PMC3655468

[ref60] Strekalova H , Buhmann C, Kleene R, Eggers C, Saffell J, Hemperly J, Weiller C, Muller-Thomsen T, Schachner M. Elevated levels of neural recognition molecule L1 in the cerebrospinal fluid of patients with Alzheimer disease and other dementia syndromes. Neurobiol Aging. 2006:27(1):1–9. 16298234 10.1016/j.neurobiolaging.2004.11.013

[ref61] Stummeyer K , Dickmanns A, Mühlenhoff M, Gerardy-Schahn R, Ficner R. Crystal structure of the polysialic acid-degrading endosialidase of bacteriophage K1F. Nat Struct Mol Biol. 2005:12(1):90–96. 15608653 10.1038/nsmb874

[ref62] Suzuki M , Suzuki M, Nakayama J, Suzuki A, Angata K, Chen S, Sakai K, Hagihara K, Yamaguchi Y, Fukuda M. Polysialic acid facilitates tumor invasion by glioma cells. Glycobiology. 2005:15(9):887–894. 15872150 10.1093/glycob/cwi071

[ref63] Tajik A , Phillips KL, Nitz M, Willis LM. A new ELISA assay demonstrates sex differences in the concentration of serum polysialic acid. Anal Biochem. 2020:600:113743. 32325083 10.1016/j.ab.2020.113743

[ref64] Tanaka F , Otake Y, Nakagawa T, Kawano Y, Miyahara R, Li M, Yanagihara K, Nakayama J, Fujimoto I, Ikenaka K, et al. Expression of polysialic acid and STX, a human polysialyltransferase, is correlated with tumor progression in non-small cell lung cancer. Cancer Res. 2000:60(11):3072–3080. 10850459

[ref65] Tyanova S , Temu T, Sinitcyn P, Carlson A, Hein MY, Geiger T, Mann M, Cox J. The Perseus computational platform for comprehensive analysis of (prote)omics data. Nat Methods. 2016:13(9):731–740. 27348712 10.1038/nmeth.3901

[ref66] Varea E , Guirado R, Gilabert-Juan J, Marti U, Castillo-Gomez E, Blasco-Ibanez JM, Crespo C, Nacher J. Expression of PSA-NCAM and synaptic proteins in the amygdala of psychiatric disorder patients. J Psychiatr Res. 2012:46(2):189–197. 22099865 10.1016/j.jpsychires.2011.10.011

[ref67] Wang T , Voglmeir J. PNGases as valuable tools in glycoprotein analysis. Protein Pept Lett. 2014:21(10):976–985. 24975665 10.2174/0929866521666140626111237

[ref68] Werneburg S , Buettner FF, Erben L, Mathews M, Neumann H, Muhlenhoff M, Hildebrandt H. Polysialylation and lipopolysaccharide-induced shedding of E-selectin ligand-1 and neuropilin-2 by microglia and THP-1 macrophages. Glia. 2016:64(8):1314–1330. 27159043 10.1002/glia.23004

[ref69] Yang SY , Huh IS, Baek JH, Cho EY, Choi MJ, Ryu S, Kim JS, Park T, Ha K, Hong KS. Association between ST8SIA2 and the risk of schizophrenia and bipolar I disorder across diagnostic boundaries. PLoS One. 2015:10(9):e0139413. 26418860 10.1371/journal.pone.0139413 PMC4587961

[ref70] Yoshimi K , Ren YR, Seki T, Yamada M, Ooizumi H, Onodera M, Saito Y, Murayama S, Okano H, Mizuno Y, et al. Possibility for neurogenesis in substantia nigra of parkinsonian brain. Ann Neurol. 2005:58(1):31–40. 15912513 10.1002/ana.20506

[ref71] Yu CC , Huang LD, Kwan DH, Wakarchuk WW, Withers SG, Lin CC. A glyco-gold nanoparticle based assay for alpha-2,8-polysialyltransferase from Neisseria meningitidis. Chem Commun (Camb). 2013:49(86):10166–10168. 24051967 10.1039/c3cc45147j

