# Supplementary material for: Site-specific immobilization of the endosialidase reveals QSOX2 is a novel polysialylated protein
Source: Glycobiology. 2024 Mar 15;34(5):cwae026. doi: 10.1093/glycob/cwae026 (PMC11031136; doi:10.1093/glycob/cwae026)
Supplement: Supplemental_v4_cwae026 [file supplemental_v4_cwae026.docx]

Supplemental information

**Site-specific immobilization of the endosialidase reveals QSOX2 is a novel polysialylated protein**

Carmanah Hunter*^1^, Tahlia Derksen^1^*, Sogand Makhsous^1^, Matt Doll^1^, Samantha Rodriguez Perez^1^, Nichollas E. Scott^2^, Lisa M. Willis^1,3^

* Authors contributed equally to the work

**Supplementary table I. Protein level analysis of NK-92 samples.** The Maxquant search results for affinity enrichment experiments of independent replicates (n=4 per group) with t-tests undertaken in Perseus are provided. For each identified protein, the log_2_ LFQ protein values, if the protein was identified by matching or by MS/MS information, the T-test information including the -log_10_(*p*‑value), difference in the mean between the groups and if the resulting *p*-values are below the multiple hypothesis corrected *p*‑values (permutation-based false discovery rate of 0.05) are provided.

**Supplementary table II. Protein level analysis of human serum.** The Maxquant search results for affinity enrichment experiments of independent replicates (n=4 per group) with t-tests undertaken in Perseus are provided. For each identified protein, the log_2_ LFQ protein values, if the protein was identified by matching or by MS/MS information, the T-test information including the -log_10_(*p*‑value), difference in the mean between the groups and if the resulting *p*-values are below the multiple hypothesis corrected *p*‑values (permutation-based false discovery rate of 0.05) are provided.

**Supplementary table III. Protein level analysis of MCF-7 samples.** The Maxquant search results for affinity enrichment experiments of independent replicates (n=4 per group) with t-tests undertaken in Perseus are provided. For each identified protein, the log_2_ LFQ protein values, if the protein was identified by matching or by MS/MS information, the T-test information including the -log_10_(*p*‑value), difference in the mean between the groups and if the resulting *p*-values are below the multiple hypothesis corrected *p*‑values (permutation-based false discovery rate of 0.05) are provided.


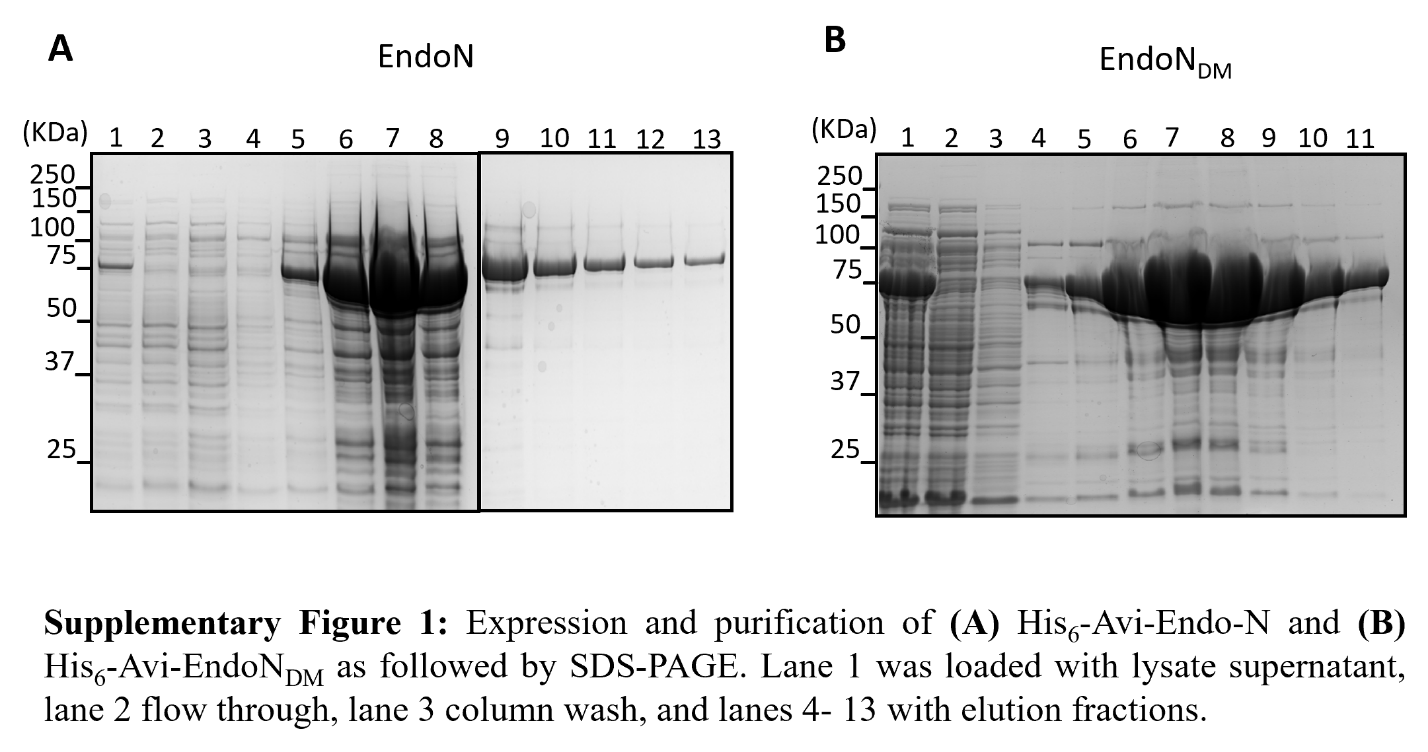


Figure S1. Expression and purification of (A) His_6_-Avi-EndoN and (B) His_6_-Avi-EndoN_DM_ by immobilized metal affinity chromatography, as followed by SDS-PAGE. Lane 1 contains the supernatant after centrifugation of the lysate, lane 2 contains the flow-through, lane 3 contains the wash, and lanes 4 – 13 contain the elution fractions.


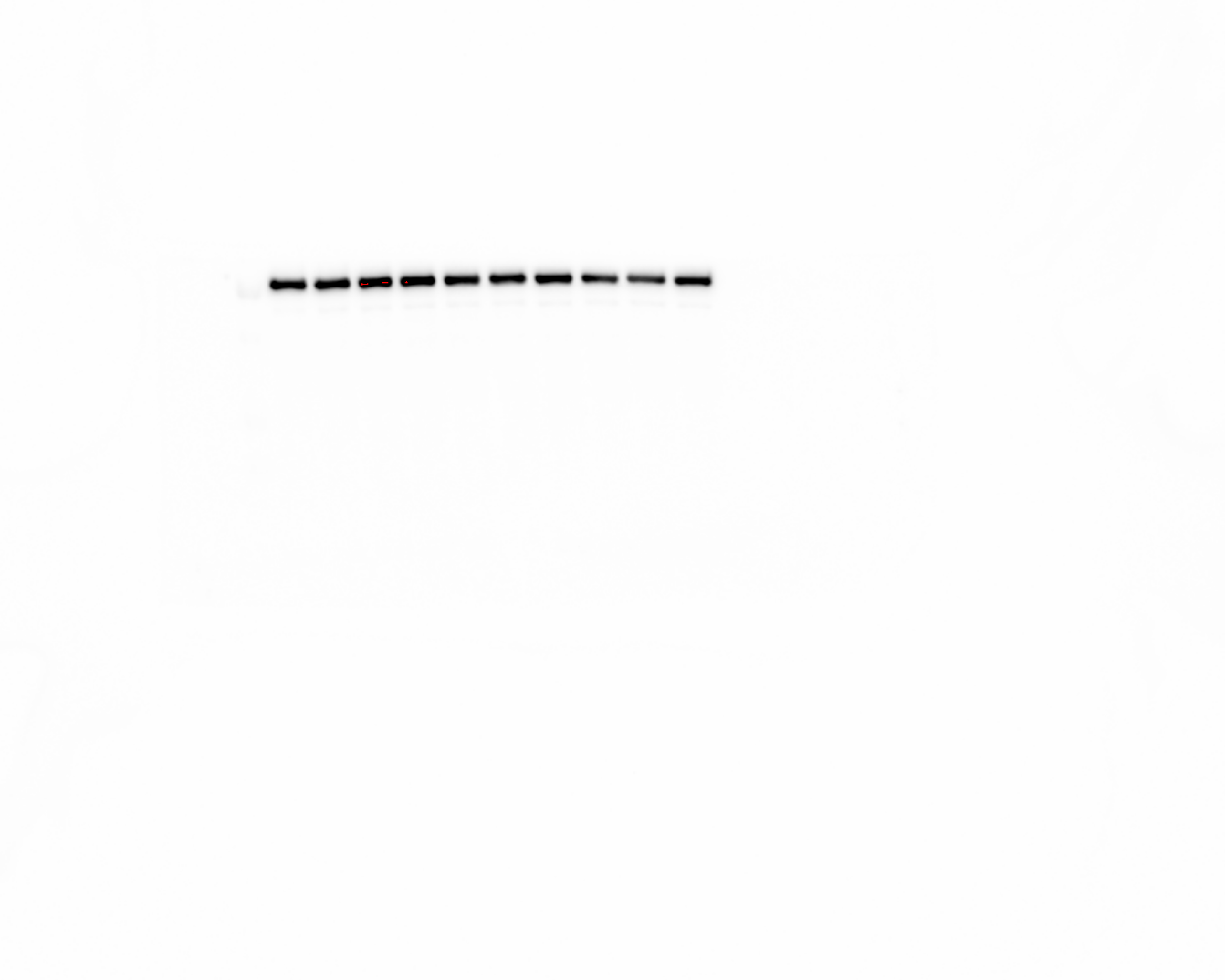

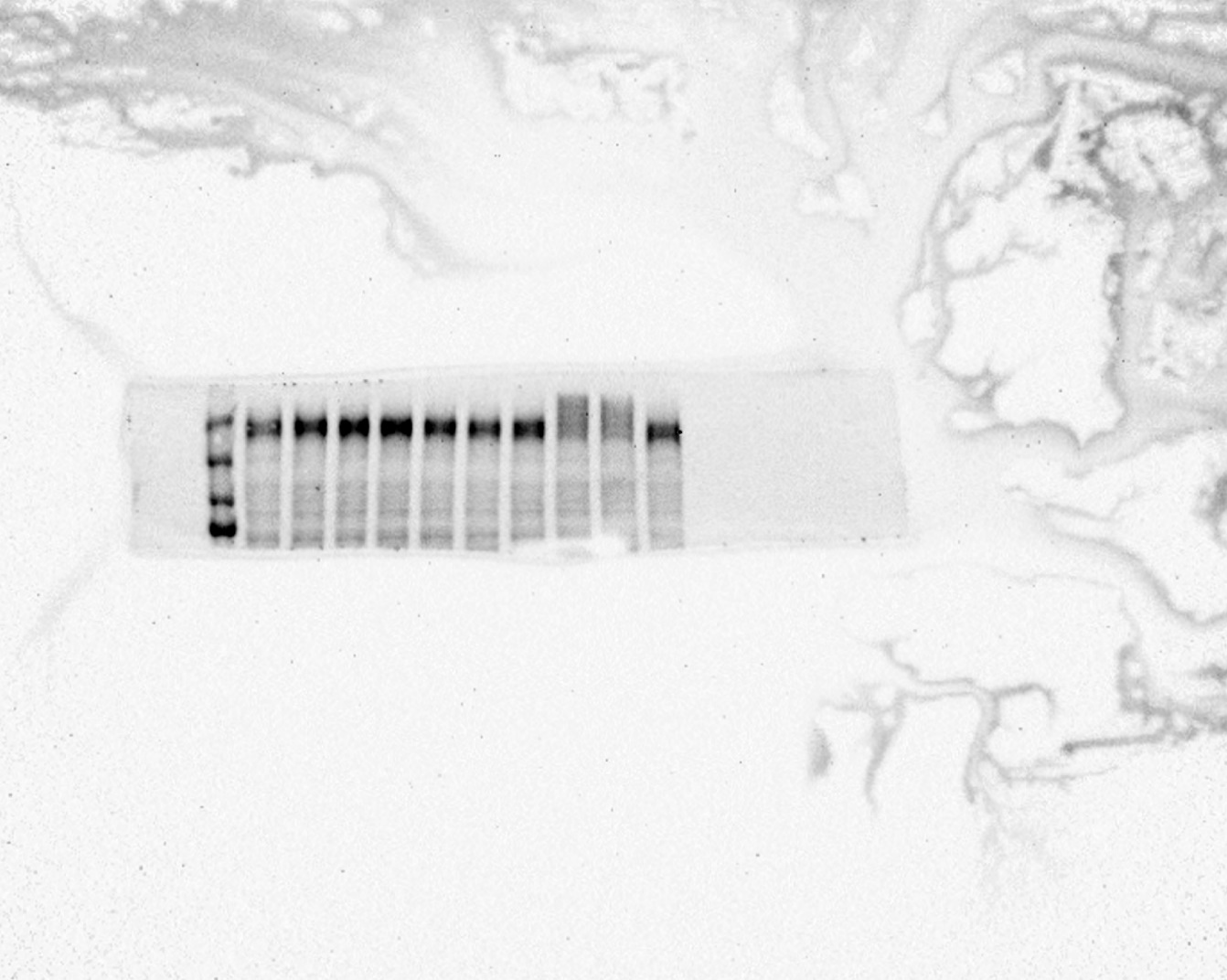

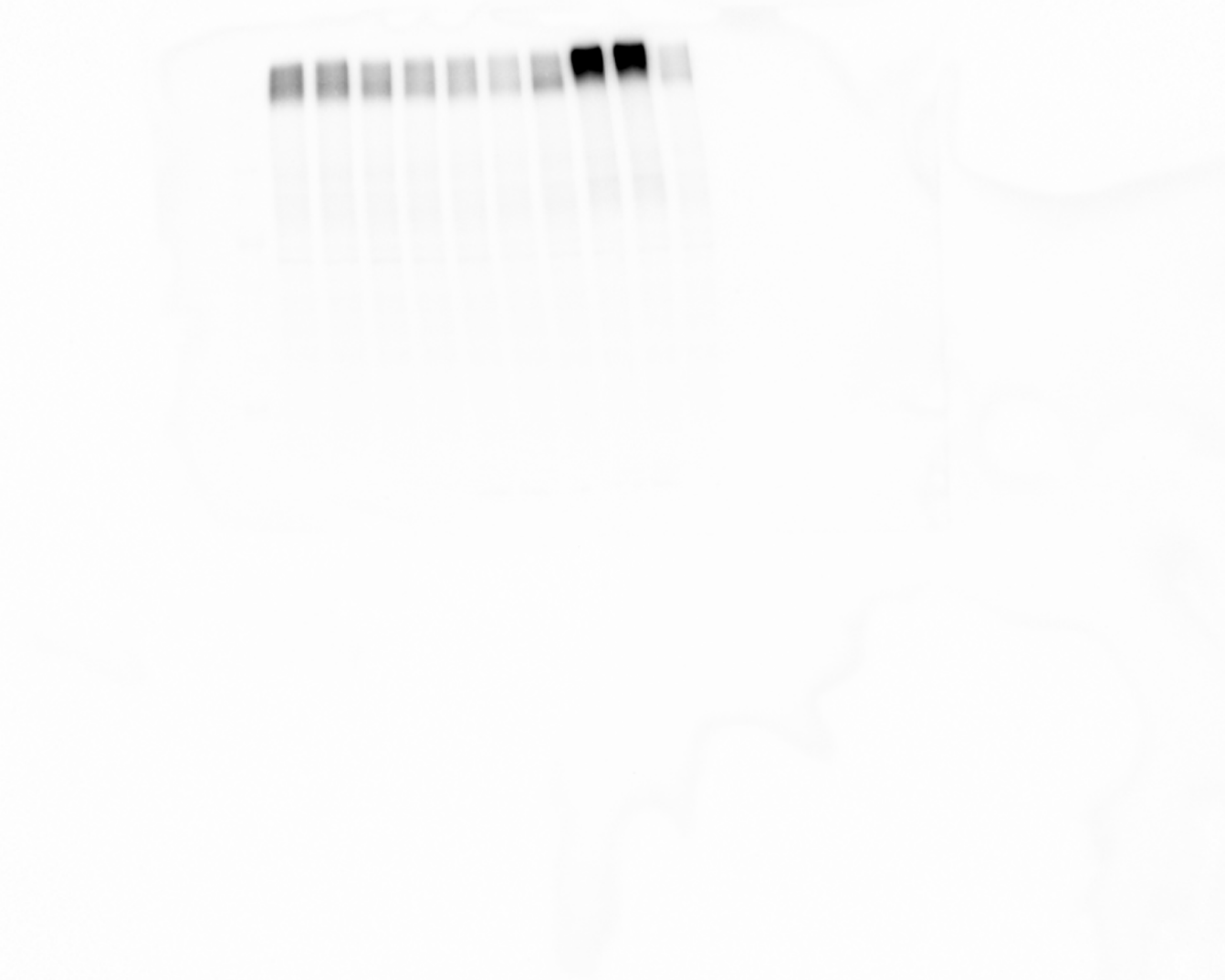


PolySia

Tubulin

NCAM

Figure S2. Immunoblots from figure 2 showing full membranes.


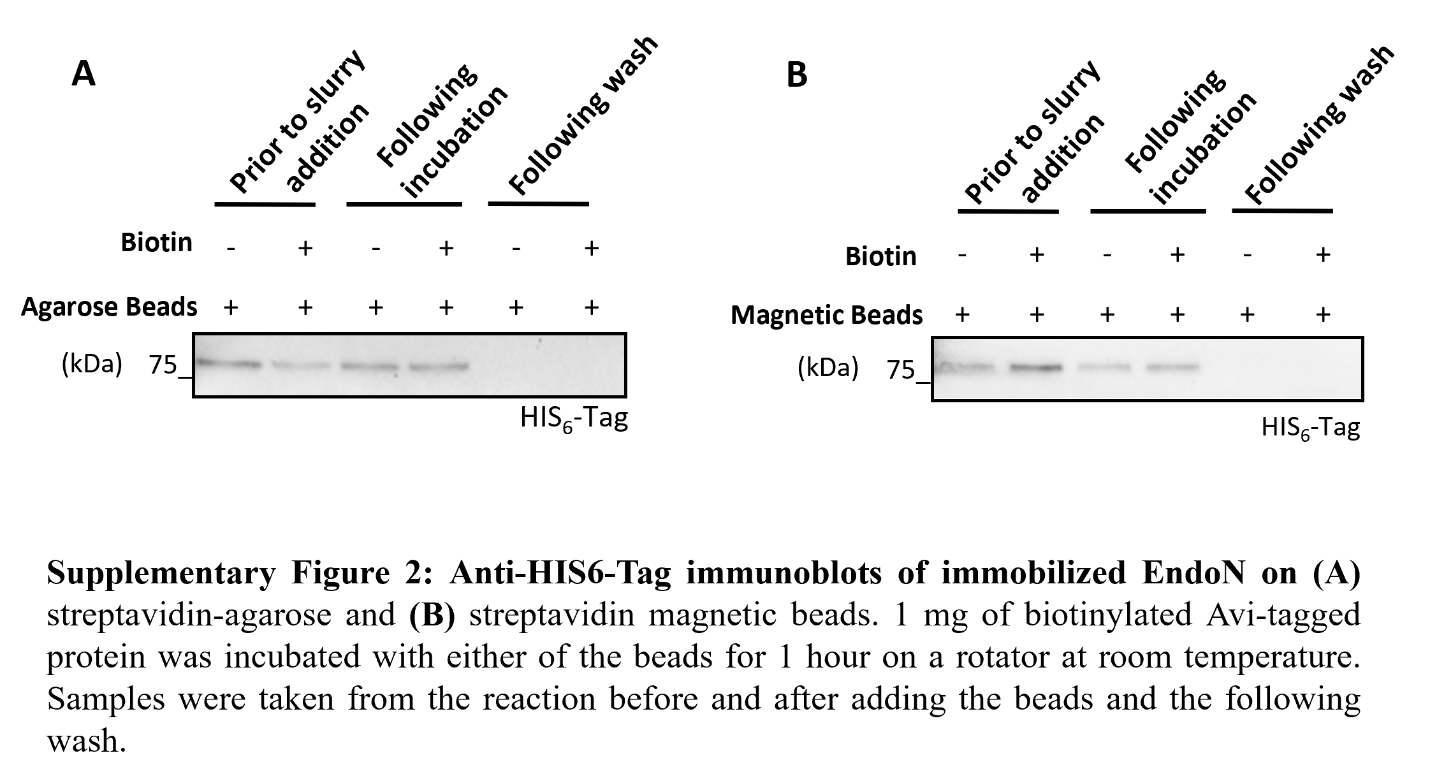


Figure S3. α-His immunoblots of immobilized EndoN on (A) streptavidin-agarose and (B) streptavidin coated magnetic beads. 1 mg of biotinylated Avi-tagged protein was incubated with beads for 1 h at room temperature with rotation. Samples were taken from the reaction before and after adding the beads and following the wash.


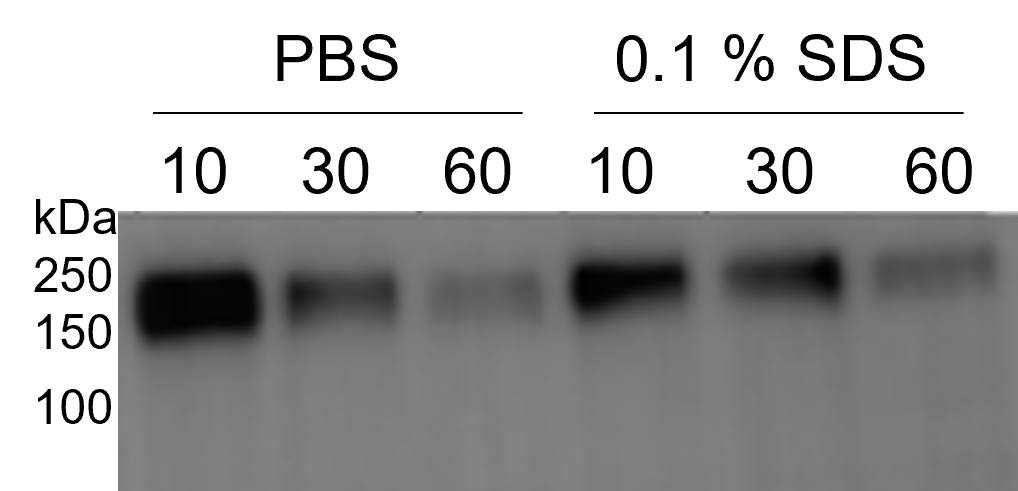


Figure S4. Washing immobilized EndoN with SDS does not substantially reduce its polySia hydrolysis activity. Immunoblot of NK-92 cell lysates treated with immobilized EndoN.

**
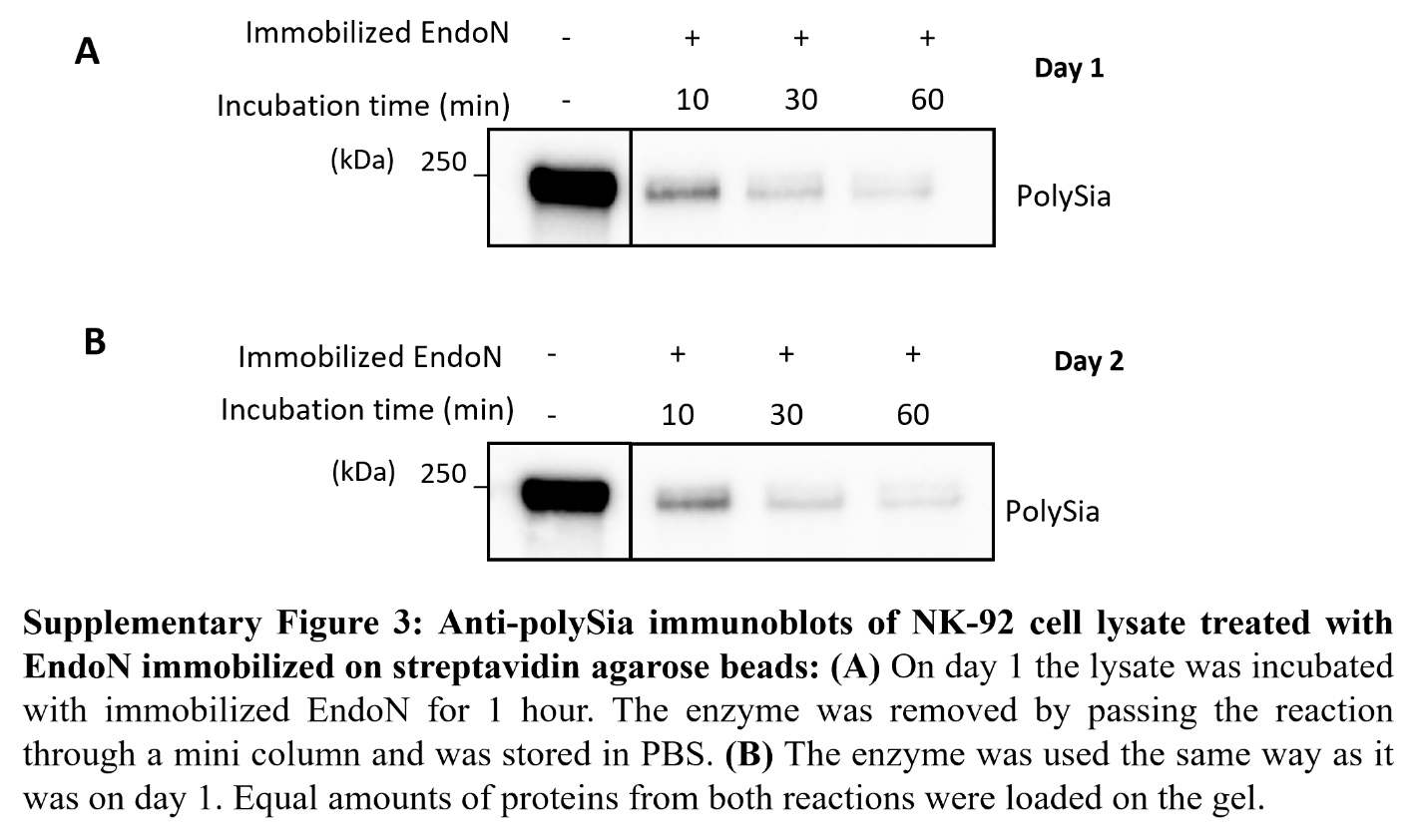
**

Figure S5. Immobilized EndoN is stable. Immobilized EndoN was incubated with NK-92 cell lysates, then recovered, washed several times with 0.1 % SDS followed by PBS, stored at 4 °C overnight, and then incubated with more NK-92 lysate. α-PolySia immunoblots from the experiment on the first day (A) and the second day (B) show similar levels of polySia hydrolysis activity.


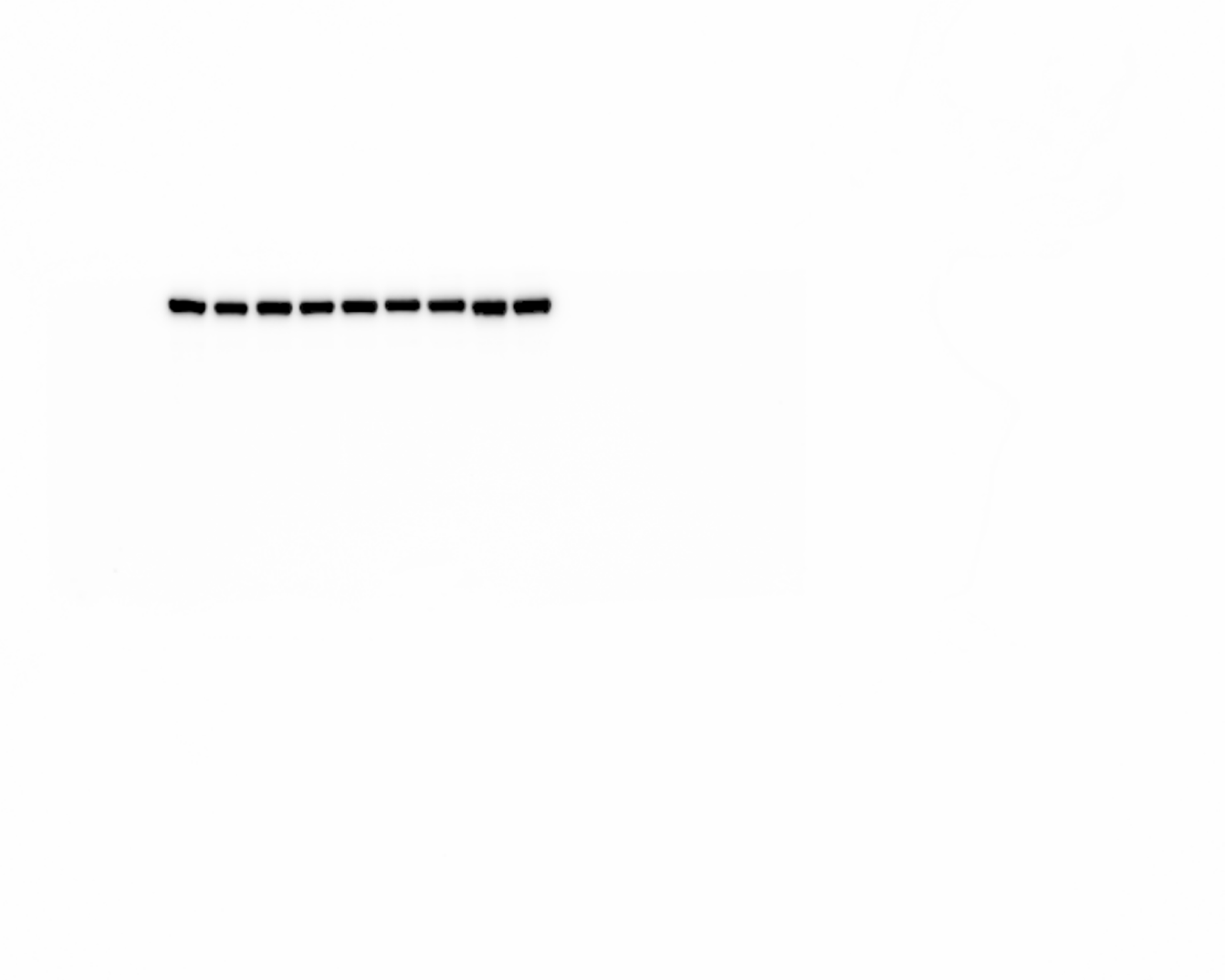

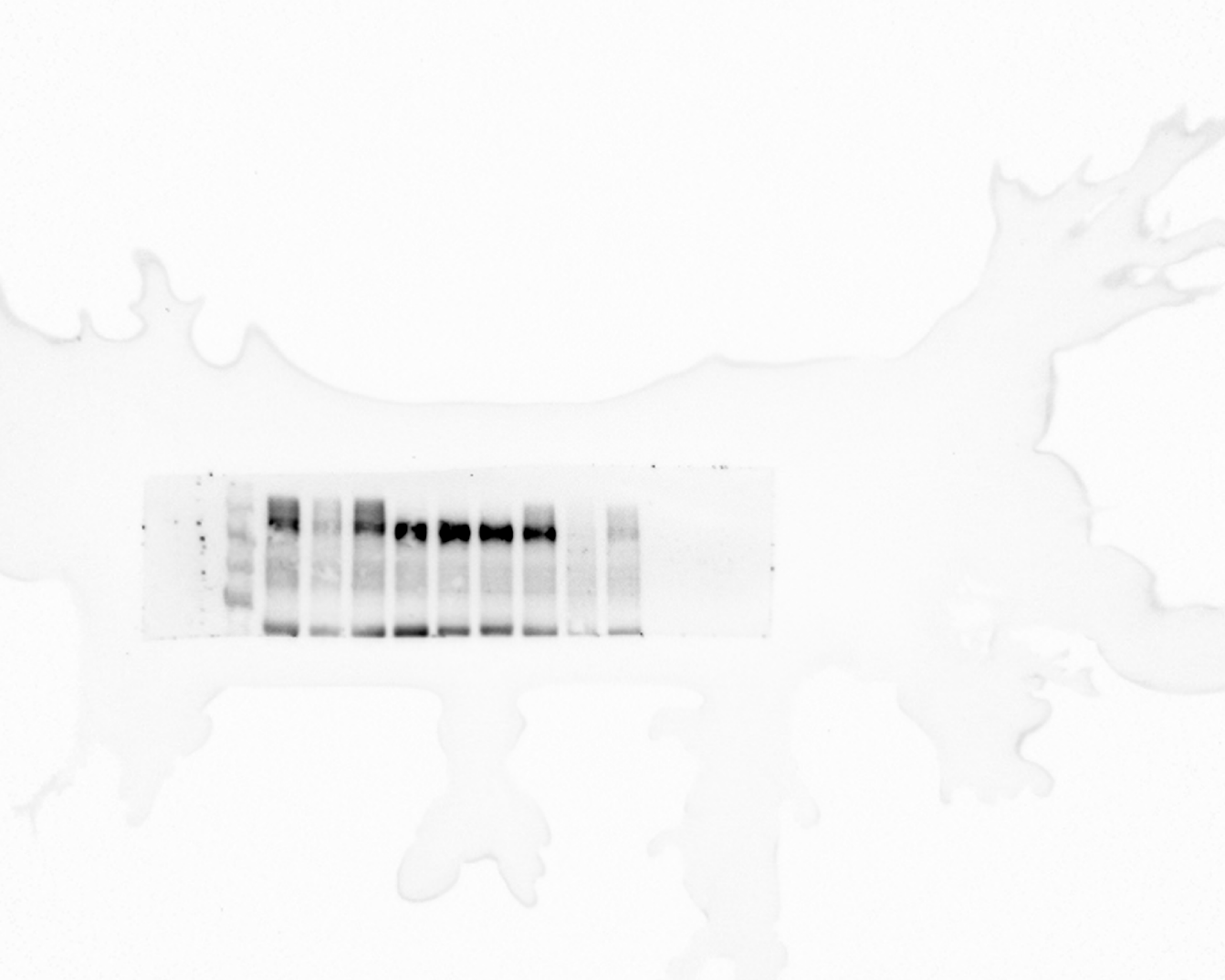

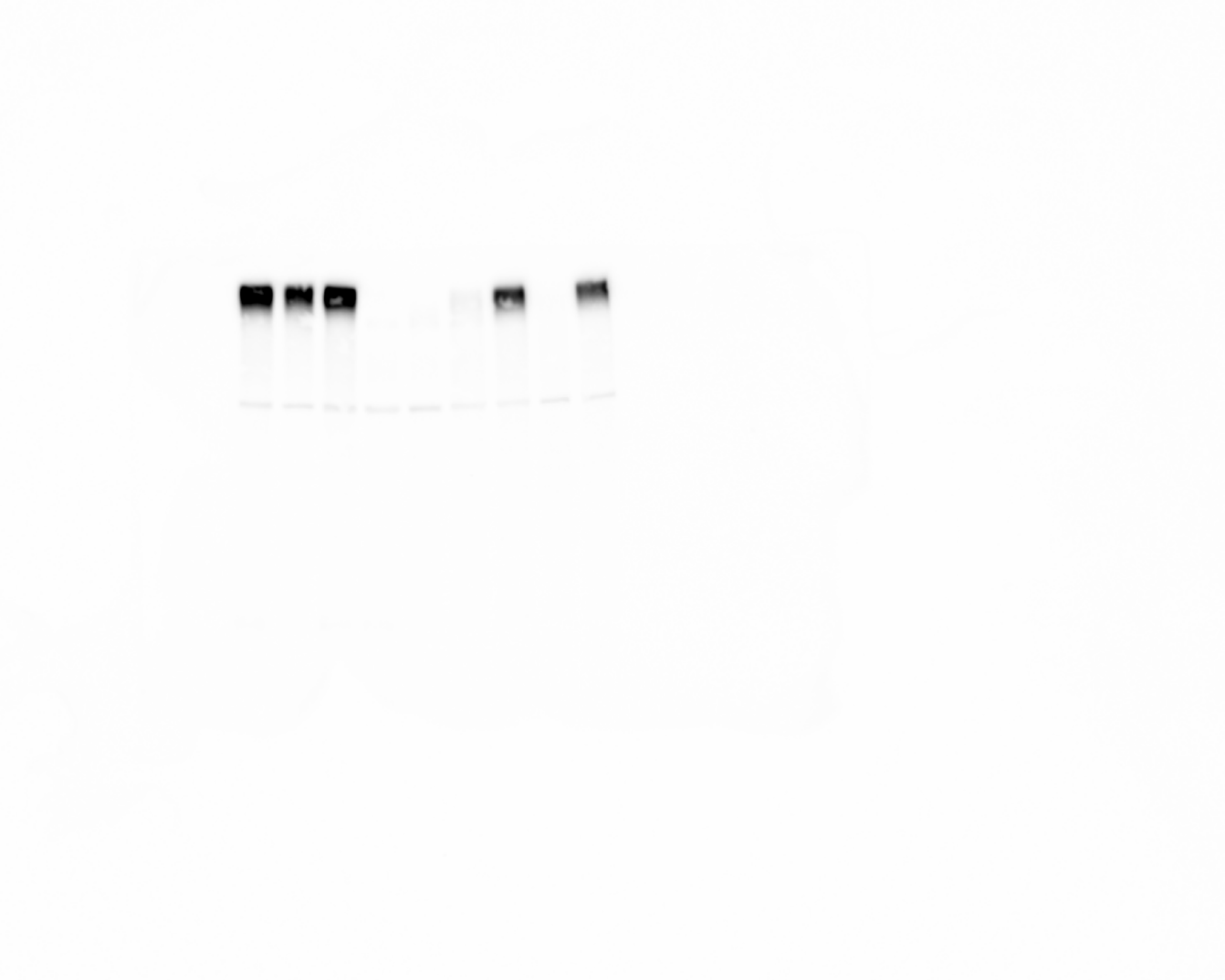


Tubulin

PolySia

NCAM

Figure S6. Immunoblots from figure 3.1 showing full membranes.

**
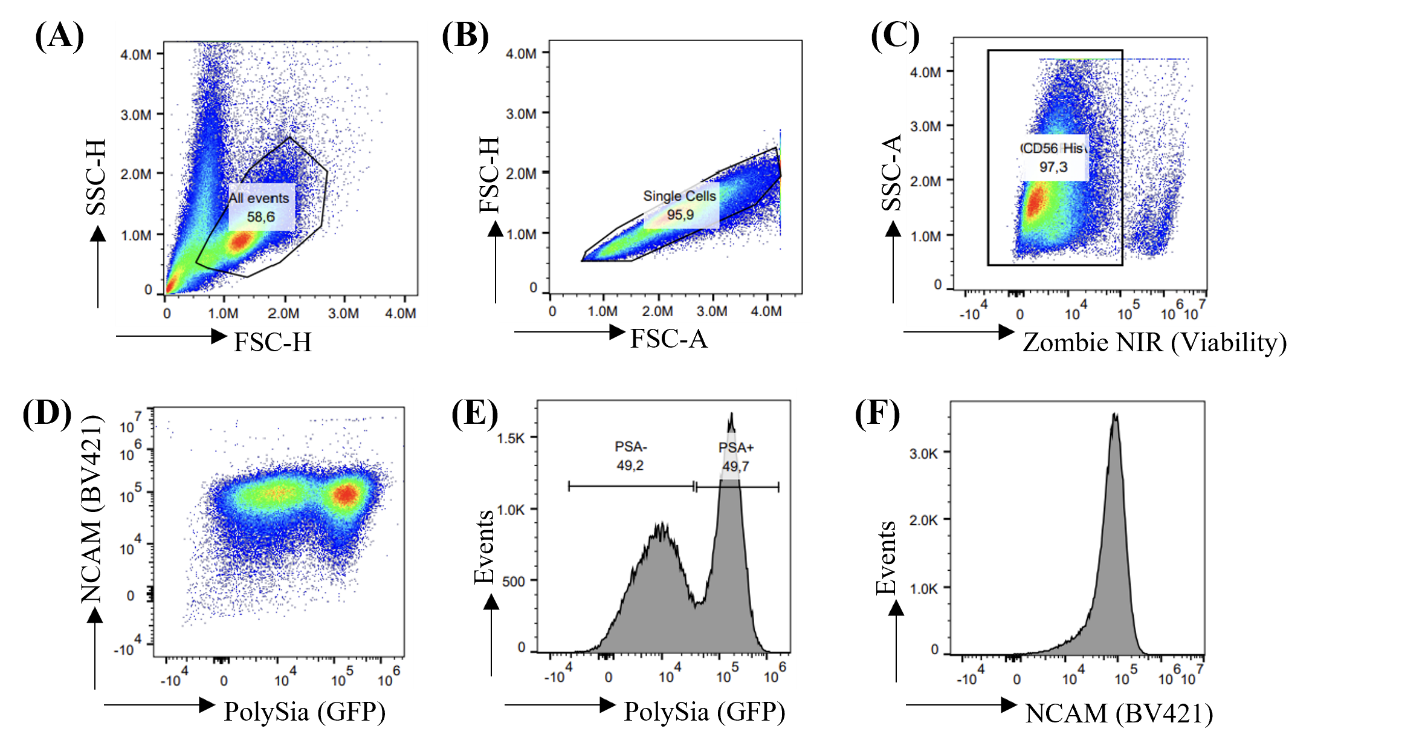
**

Figure S7. Flow cytometry gating strategy for visualizing polySia and NCAM on NK-92 cells. Vials containing the same amount of untreated and immobilized EndoN treated NK-92 cells were mixed, incubated for 1 hour at 37 °C, and followed by flow cytometry: (A) Cells were gated on forward vs. side scatter, (B) single cells were selected, (C) within the single cells population, the live cells were gated and (D, E, F) live cells were analyzed for polySia and NCAM expression.


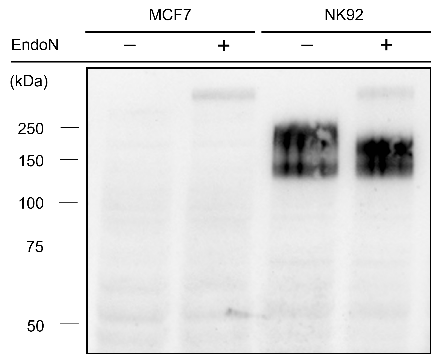


Figure S8. MCF-7 cells do not express NCAM. α-NCAM immunoblot of lysates from MCF-7 and NK92 (positive control).


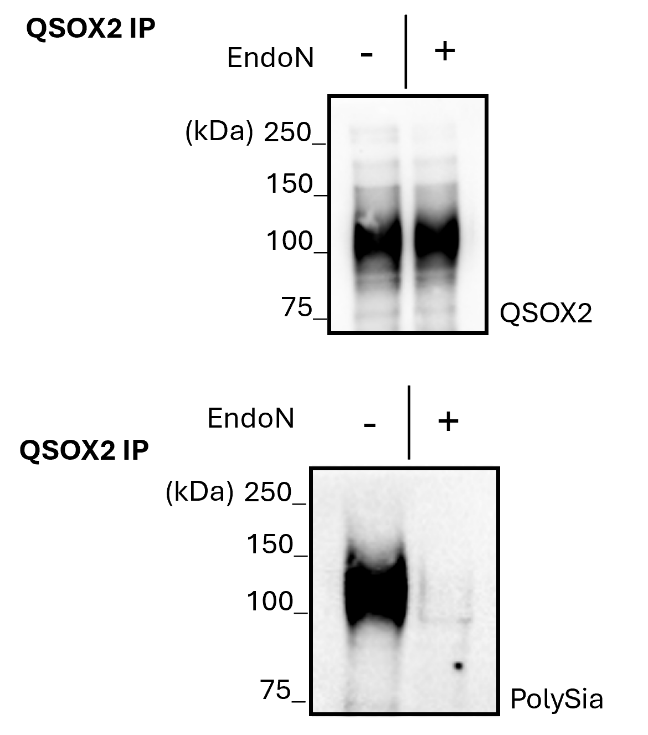

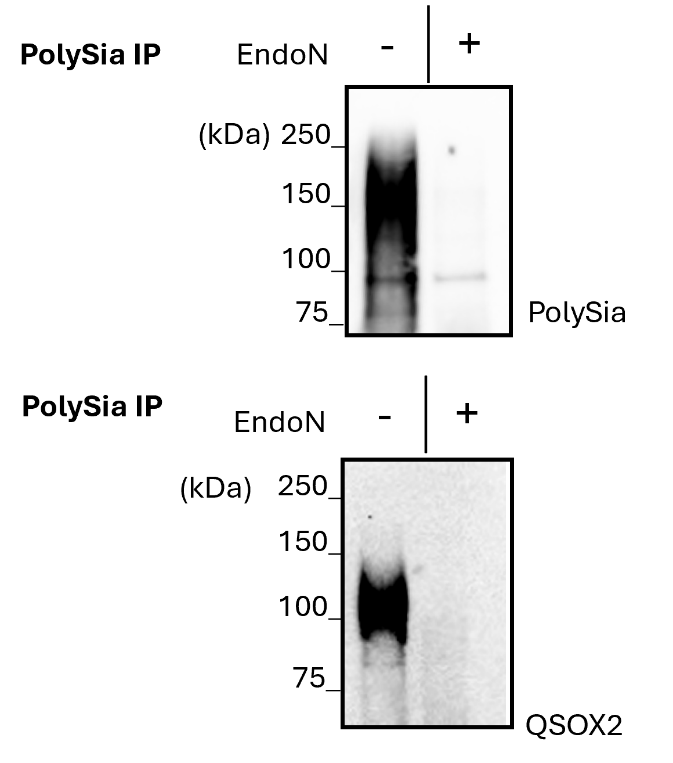


Figure S9. Reciprocal immunoprecipitation of polysialylated QSOX2. MCF-7 lysates were pretreated with PBS or EndoN (-/+ EndoN) before immunoprecipitation followed by blotting.
